# Biodegradable Acoustic Targeting for Ultrasound‐Supported Gene Therapy (BATUS) in Glioblastoma

**DOI:** 10.1002/advs.76336

**Published:** 2026-06-29

**Authors:** Gulsah Erel‐Akbaba, Jinyoung Park, Achal Duhoon, Cao Thuy Giang Nguyen, Nitu Bhaskar, Sumanta Kumar Karan, Hasan Akbaba, Parbeen Singh, Thi Bao Tram Tran, Yuhui Zhu, Zhiming Li, I'jaaz Muhammad, Xiaojun Zhang, Hoang Quan Truong, A. Daniel Davidar, Elizabeth Schmitzer, Vikas N. Vattipally, Angelica F. Lopez, Patrick Kramer, Victor M. Quiroz, Ben Bykov, Claire Hao, Amir Manbachi, Nicholas Theodore, Joshua C. Doloff, Thanh D. Nguyen

**Affiliations:** ^1^ Polymer Program Institute of Materials Science University of Connecticut Storrs Connecticut USA; ^2^ Department of Pharmaceutical Biotechnology Faculty of Pharmacy Izmir Katip Celebi University Izmir Turkey; ^3^ Department of Biomedical Engineering University of Connecticut Storrs Connecticut USA; ^4^ Department of Mechanical Engineering University of Connecticut Storrs Connecticut USA; ^5^ Center for Clean Energy Engineering University of Connecticut Storrs CT USA; ^6^ Ege University Vaccine Development Application and Research Center Izmir Turkey; ^7^ Department of Pharmaceutical Biotechnology Faculty of Pharmacy Ege University Izmir Turkey; ^8^ Department of Neurosurgery Johns Hopkins School of Medicine Baltimore Maryland USA; ^9^ Department of Biomedical Engineering Johns Hopkins School of Medicine Baltimore Maryland USA; ^10^ Department of Materials Science and Engineering Johns Hopkins University Baltimore Maryland USA; ^11^ Department of Oncology Division of Cancer Immunology Sidney‐Kimmel Comprehensive Cancer Center Bloomberg∼Kimmel Institute for Cancer Immunotherapy Johns Hopkins University Baltimore Maryland USA

**Keywords:** biodegradable ultrasound transducer, blood–brain barrier opening, glioblastoma immunotherapy, piezoelectric biomaterials, ultrasound‐mediated gene delivery

## Abstract

Glioblastoma (GBM) remains one of the most challenging brain malignancies due to the restrictive nature of the blood–brain barrier (BBB), which severely limits effective drug and gene delivery. To overcome this, we introduce Biodegradable Acoustic Targeting for Ultrasound‐Supported Gene Therapy (BATUS), a modular platform that enables safe, repeated, and targeted gene delivery to the brain. BATUS integrates an implantable, fully biodegradable glycine‐based ultrasound (US) transducer, surgically placed via craniotomy for precise BBB opening, along with customizable peptide‐targeted liposomal gene carriers. This system combines US‐mediated transient BBB disruption with ligand‐directed cellular targeting to enable efficient delivery across both vascular and cellular barriers. As a proof‐of‐concept, BATUS was evaluated using tLyp1‐functionalized liposomes carrying PD‐L1‐targeting siRNA for GBM immunotherapy: in vitro evaluations confirmed efficient siRNA entrapment, stability, and specific cellular uptake, while in vivo studies in orthotopic GL261 GBM mouse models showed enhanced BBB permeability, significant PD‐L1 silencing, reduced tumor growth, and prolonged survival. Crucially, BATUS exhibited a favorable safety profile, with no detectable local or systemic toxicity in animal models. By providing a modular framework where targeting ligands and genetic cargo are easily adaptable, BATUS is established as a strategy for precision gene delivery across the BBB, as demonstrated here for GBM.

## Abstract 2

Biodegradable Acoustic Targeting for Ultrasound‐Supported Gene Therapy (BATUS) enables safe, repeated, and localized blood–brain barrier opening using an implantable ultrasound transducer, combined with peptide‐targeted liposomal gene delivery. In the glioblastoma model, this approach enhances delivery efficiency, achieves effective PD‐L1 silencing, suppresses tumor growth, and prolongs survival, highlighting a versatile and translational strategy for precision gene delivery to the brain.

## 1 Introduction

Brain disorders, particularly aggressive malignancies such as Glioblastoma (GBM), present formidable treatment challenges due to the blood‐brain barrier (BBB), characterized by tight junctions between endothelial cells, which restrict the delivery of most therapeutic agents to the brain [1]. While various neurological diseases are affected by BBB limitations, GBM represents one of the most challenging indications due to its highly invasive nature and poor prognosis, making it a relevant model for evaluating BBB‐targeted delivery strategies [2].

A range of strategies, including adjuvant application, lipidization, and osmotic disruption, have been explored to modulate the BBB; however, focused ultrasound (FUS), particularly when combined with intravenously administered microbubbles (MBs), represents one of the most effective and clinically advanced approaches for enhanced local delivery of drugs to precise areas of the brain [3]. The oscillation of MBs under US induces mechanical stress on vascular walls, leading to a temporary and reversible increase in BBB permeability [4]. However, clinical translation of extracorporeal FUS systems remains limited due to skull‐induced acoustic attenuation (>90%), the need for high energy input, and reliance on real‐time MRI guidance [5, 6]. Implantable US transducers have emerged as an alternative approach to enable repeated and localized BBB modulation with lower energy requirements [7]. While implantable US systems require surgical placement, this consideration should be interpreted within the clinical context of high‐grade brain tumors such as GBM, where surgical intervention is routinely performed as part of standard care. In this setting, implantable devices may be incorporated into existing surgical workflows. Furthermore, approaches enabling repeated and localized BBB modulation may offer advantages in cases where non‐invasive strategies are insufficient to achieve effective drug delivery. For example, the SonoCloud system has demonstrated the feasibility of repeated BBB opening in clinical settings. However, currently available devices are typically composed of non‐degradable piezoelectric materials (e.g., lead zirconate titanate), requiring surgical removal and raising long‐term safety concerns [8]. This perspective motivates the development of implantable yet biodegradable systems designed to minimize long‐term burden while enabling controlled therapeutic delivery.

Our group has recently reported a fully biodegradable, flexible, easy‐to‐use US transducer made of a stable, highly piezoelectric nanofiber film of glycine crystals embedded in a polycaprolactone polymeric matrix, enabling controlled and repeatable BBB opening without the need for device removal [9]. While this system has demonstrated enhanced delivery of chemotherapeutic agents in both healthy brains and orthotopic GBM models, conventional drugs such as paclitaxel lack targeting specificity and may induce systemic toxicity, limiting their broader applicability [10]. Effective delivery across the BBB requires overcoming multiple sequential biological barriers, including vascular transport, BBB penetration, and cellular uptake within the brain parenchyma [11]. Building on this, we introduce Biodegradable Acoustic Targeting for US‐Supported Gene Therapy (BATUS) as an advanced platform technology that integrates the biodegradable transducer with customizable, targeted liposomes to achieve efficient and selective gene delivery.

GBM was selected in this study as a proof‐of‐concept model due to its aggressive nature, poor prognosis, and limited treatment options, with median survival remaining approximately 12–15 months despite current therapies and fewer than 5% of patients surviving beyond five years post‐diagnosis [12, 13, 14, 15]. Unlike traditional chemotherapeutics, immunotherapy activates immune cells to attack tumors and generates lasting immune memory to prevent relapse. Despite the development of numerous immunotherapeutic agents, treatment efficacy in GBM is limited not only by the BBB but also by intrinsic low immunogenicity and adaptive immune resistance driven by checkpoint overexpression and infiltration of immunosuppressive cells [16]. Immune‐checkpoint inhibitors targeting the PD‐1/PD‐L1 pathway are especially promising, as PD‐L1 is often overexpressed in tumors and the myeloid compartment, which contributes to T‐cell exhaustion [14, 17, 18, 19]. PD‐L1 expression in GBM is highly dynamic and can be significantly upregulated in response to radiotherapy, IFN‐γ signaling, hypoxia, and recurrent disease. Several recent studies show that PD‐L1 can be induced in both tumor and myeloid compartments under treatment pressure, contributing to therapeutic resistance. Current immune checkpoint inhibitor‐based therapies rely on antibody‐based competitive inhibition of immunosuppressive molecules [20]. However, immunotherapeutic antibodies also often induce adverse local and systemic immune reactions [21]. To circumvent these limitations while retaining precision, we employed siRNA‐mediated PD‐L1 silencing as a safer and more specific alternative, delivered via the BATUS platform.

Despite its promise, siRNA‐mediated gene therapy is hampered by poor cellular uptake, rapid degradation, and fast clearance [22, 23]. Liposomes represent an attractive non‐viral delivery system due to their biocompatibility and tunable properties, and their targeting efficiency can be enhanced through conjugation with tumor‐penetrating peptides such as tLyp1(CGNKRTR) [24], which binds neuropilin‐1 (NRP‐1) overexpressed in GBM, further improves tumor penetration and specificity [25, 26, 27, 28]. Notably, the BBB in GBM is heterogeneous and may exhibit regions of partial disruption, particularly within the tumor core, which can contribute to nanoparticle accumulation [29]. However, this permeability is spatially variable and often insufficient for uniform and effective therapeutic delivery. Therefore, distinguishing US‐mediated BBB opening from tumor‐associated vascular leakiness remains important for accurately interpreting delivery efficiency. To date, no platform has combined non‐viral, peptide‐targeted gene delivery with safe, repeated, implantable US‐mediated BBB opening [30, 31, 32, 33, 34].

Here, we present BATUS, a biodegradable acoustic targeting approach that integrates our fully biodegradable glycine‐based piezoelectric US transducer with customizable, peptide‐targeted liposomal gene carriers to achieve safe, repeated, and precise gene delivery across the BBB. This system combines US‐mediated transient BBB opening with ligand‐directed targeting to facilitate delivery across both vascular and cellular barriers. As proof of concept, we demonstrate the delivery of tLyp1‐functionalized liposomes carrying PD‐L1‐targeting siRNA in orthotopic GL261 GBM models, resulting in effective gene silencing, tumor regression, and prolonged survival with a favorable safety profile. While this study focuses on GBM, the modular nature of the system suggests potential adaptability to other neurological applications.

## 2 Results and Discussion

### 2.1 Development of Targeted Liposomes for Gene Delivery via the BATUS Platform

**FIGURE 1:**
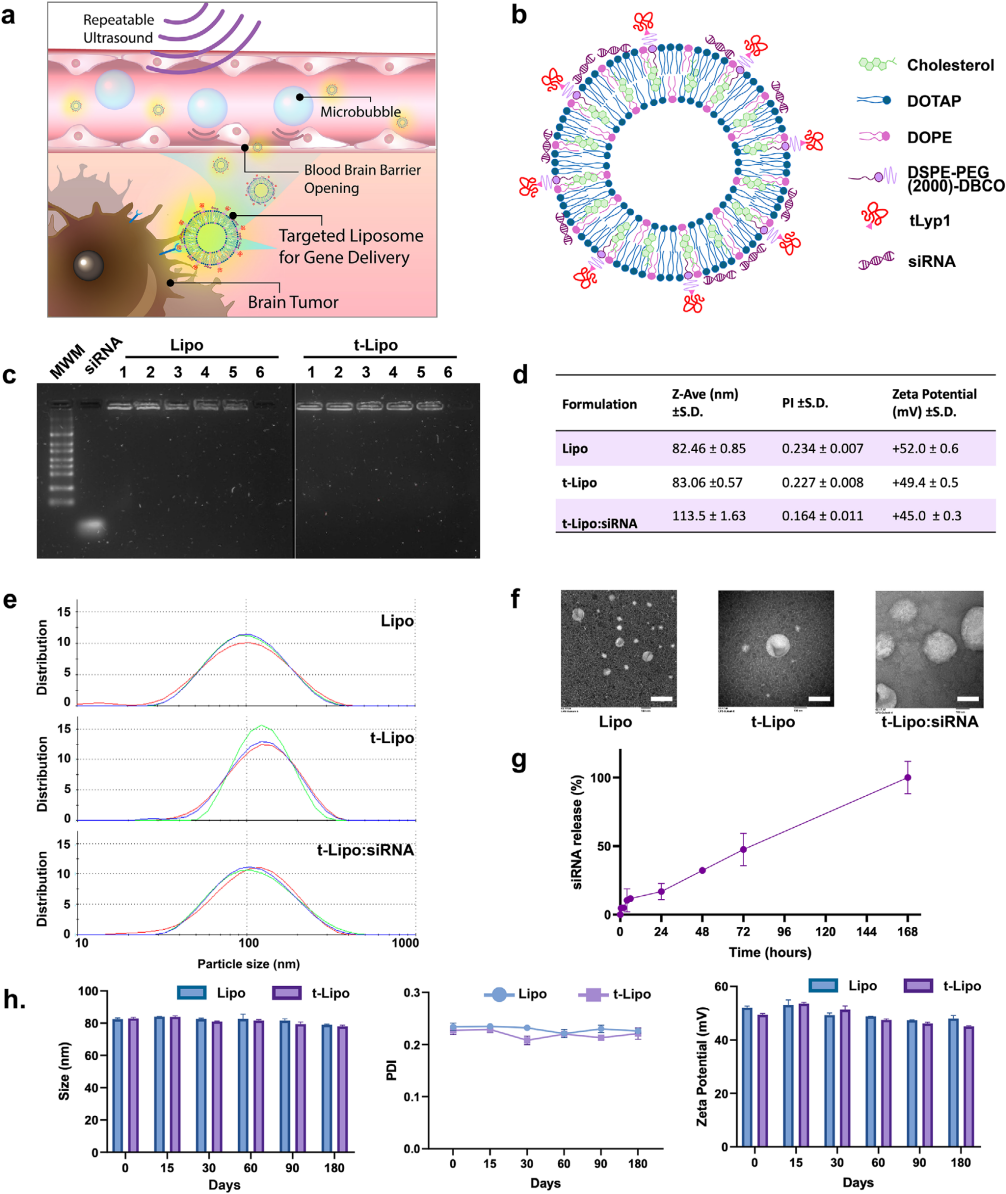
Comprehensive illustration of the BATUS platform and targeted liposome (t‐Lipo) development for gene delivery to brain tumors. (a) Schematic representation of the BATUS platform showing the use of repeatable US from a biodegradable device with microbubbles to open the blood‐brain barrier (BBB), facilitating the repeated delivery of targeted liposomes for gene therapy to brain tumors. (b) Diagram of the liposome composition, including DOTAP, DOPE, cholesterol, DSPE‐PEG(2000)‐DBCO, tLyp1 peptide, and siRNA, illustrating the structure of the targeted liposome (t‐Lipo) formulation. (c) Gel retardation assay demonstrating siRNA binding to Lipo and t‐Lipo at various N+/P‐ ratios (1:1, 2:1, 3:1, 4:1, 5:1), showing complete retardation starting at a 1:1 ratio. Lane 6 is only Lipo (d) Table summarizing the physicochemical properties (particle size, PDI, and zeta potential) of Lipo, t‐Lipo, and t‐Lipo:siRNA formulations as measured by DLS. (e) Particle size distribution curves for Lipo, t‐Lipo, and t‐Lipo:siRNA. (f) Transmission electron microscopy (TEM) images of Lipo, t‐Lipo, and t‐Lipo:siRNA. (The white scale bar is 200 nm) (g) In vitro release profile of siRNA from t‐Lipo:siRNA over 168 h. (h) Stability assessment of Lipo and t‐Lipo over 180 days at 4°C (n = 3).

The BBB is a highly selective barrier composed of endothelial cells, pericytes, and astrocytes that restricts the passage of most molecules into the brain. US, when combined with MBs, induces mechanical stress on the BBB, leading to a temporary and reversible opening. US‐mediated BBB opening allows nanoparticles to cross the barrier and accumulate in the brain parenchyma at high concentrations [35, 36]. Our newly developed technology, BATUS, employs our unique biodegradable US transducer to repeatedly open the BBB with the help of MBs to achieve gene‐based therapy (Figure 1a). In addition to opening the BBB, specifically targeting diseased cells is also important for the treatment of brain diseases [37]. For this purpose, tumor‐targeting liposomes were engineered with the targeting peptide tLyp1 to enhance their uptake by GBM cells.

In this study, liposomes (Lipo) composed of dioleoyl‐3trimethylammonium propane (DOTAP), 1,2‐Dioleoyl‐sn‐glycero‐3‐PE (DOPE), cholesterol, and DSPE‐PEG with DBCO‐binding groups were prepared using the film hydration technique. PEG‐conjugated amphiphilic lipopolymers, particularly phospholipid‐based PEG‐lipopolymers like DSPE‐PEG, play a crucial role in enhancing delivery efficiency by minimizing liposome‐induced complement activation compared to commonly used alternatives, highlighting their potential to improve the safety profile of delivery systems. Additionally, incorporating DSPE‐PEG significantly extends circulation time in the bloodstream, decreases reticuloendothelial system uptake, and boosts the bioavailability of drugs in target organs [38, 39]. A specially synthesized ligand, tLYP1 with an azide group, was chemically attached to the ‐DBCO group in the Lipo formulation through copper‐free click chemistry, resulting in the formation of the targeted liposome (t‐Lipo). Subsequently, the siRNA was electrostatically bound to the outer surface of the formulation, leading to the formation of t‐Lipo:siRNA (Figure 1b). As a control for receptor‐mediated uptake, we prepared a liposome conjugated with the scrambled tLyp1 peptide (RKGNTRC) using the same conjugation protocol, referred to as non‐t‐Lipo.

The nucleic acid binding ability of the prepared liposomes was evaluated by gel retardation assay with various N^+^/P^−^ ratios of siRNA to liposome (N^+^/P^−^, 1:1, 2:1, 3:1, 4:1, and 5:1) and revealed that both Lipo and t‐Lipo formulations have the ability to complex with siRNA. Specifically, siRNA migration was completely impeded, starting from a N^+^/P^−^ ratio of 1:1 (Figure 1c). A lower N ratio can be advantageous, as it indicates sufficient binding capacity without excessive positive charge that could lead to cytotoxicity or hinder cellular uptake [40]. Thus, a ratio of 2:1 (N^+^/P^−^) was used to prepare the Lipo:siRNA and t‐Lipo:siRNA complexes in all subsequent experiments.

Particle size, polydispersity index (PDI), and zeta potential of the formulations were assessed using dynamic light scattering (DLS). The mean diameter of Lipo was measured at 82.46 nm, which did not significantly differ from that of t‐Lipo, recorded at 83.06 nm (Figure 1d,e). In our previous studies, we hypothesized that this similarity could be attributed to the surface‐stabilizing effect of short‐chain peptides on liposomes [28, 41]. Upon complexation with siRNA, the size increased to 113.5 nm, whereas the PDI value decreased from 0.227 to 0.164, indicating that the formulation became more monodisperse (Figure 1d,e). The particle size of liposomes is critical for their efficiency in traversing the BBB, as studies suggest that nanoparticles in the range of 20–200 nm can help to increase the penetration through this barrier [42]. Transmission electron microscopy (TEM) images corroborated the particle size measurements obtained using DLS (Figure 1f). Zeta potential measurements revealed that all prepared formulations exhibited a positive surface charge (Figure 1d,e), as anticipated because of the presence of the cationic lipid DOTAP. The interaction between cationic liposomes and negatively charged nucleic acids results in a decrease in zeta potential, which can promote more uniform particle sizes. Moreover, this charge interaction leads to the formation of lipoplexes capable of traversing cellular membranes and delivering genetic material into cells [43].

The in vitro release characteristics of siRNA from t‐Lipo:siRNA complexes (2:1, N^+^/P^−^) were also investigated (Figure 1g). In the first 6h, 11.75 ± 0.87% of siRNA was released. Substantial siRNA release was observed at 48h, where the amount released reached 32.22 ± 2.4% at 48h, 42.5 ± 11.5% at 72h, and 100 ± 11.8% at 168h, respectively. These kinetics indicated a suitable release profile that allowed for the accumulation and uptake of the formulation through the BBB before releasing most of the siRNA [44]. The entrapment efficiency of the t‐Lipo:siRNA complex (2:1, N^+^/P^−^) formulation was evaluated using a RiboGreen assay. The entrapment efficiency of the t‐Lipo:siRNA complex was evaluated using a RiboGreen assay. We found that 86.5 ± 5.4% of siRNA was associated with the liposome formulation and protected from detection in the absence of formulation dissociation. Furthermore, agarose gel electrophoresis also indicated high entrapment efficiency (Figure 1c) [45]. Recent advancements in liposome technology have shown that achieving entrapment efficiencies (EE) of 80% or higher is crucial for enhancing the therapeutic efficacy and specificity of RNA‐based treatments [46]. High EE(%) protects siRNA from enzymatic degradation and facilitates its uptake by target cells, which is essential for maintaining the integrity and functionality of siRNA during systemic circulation to ensure that siRNA can be released effectively within target cells [47].

The physicochemical properties of Lipo and t‐Lipo formulations stored at 4°C were monitored over a period of 180 days (Figure 1h). The particle size and zeta potential of the Lipo formulation exhibited a slight decrease from 82.46 nm (day 0) to 79.04 nm and from 52 mV to 48 mV, respectively. Similarly, the t‐Lipo formulation showed a minor reduction in size and zeta potential, from 83.06 nm to 78.07 nm and from 49.4 mV to 45 mV, respectively, over the same period. Importantly, the zeta potential remained positive, a critical factor for preserving the siRNA binding capability of the formulation [48]. The PDI values for both formulations remained below 0.3, indicating monodispersity, with no statistically significant changes observed over the 180‐days (p > 0.05) [49]. The literature emphasizes that maintaining the stability of liposomes is crucial for preserving their integrity and functionality in gene delivery applications [50], and is influenced by various factors, with lipid composition being among the most significant. Cholesterol, utilized in our formulation, is a key component that enhances membrane stability by modulating fluidity, permeability, and membrane strength [51]. In conjunction with cationic lipids, such as DOTAP, and helper lipids like DOPE, it aids in maintaining the structural integrity of liposomes at 4°C by preventing phase transitions, thereby improving the stability and functionality of liposomes for gene delivery [52].

### 2.2 Assessment of Safety and Efficacy of Targeted Liposomes in vitro

First, the cytotoxicity of different concentrations of empty Lipo and t‐Lipo formulations was evaluated on cultured cells to demonstrate the biocompatibility of the liposomal carrier itself. The objective was to confirm that the liposome platform does not induce inherent toxicity in either U87 or GL261 cells. U87 cells were used exclusively for in vitro studies as a human GBM cell line to assess biocompatibility and gene silencing efficiency in a controlled setting, while all in vivo therapeutic evaluations were conducted in the orthotopic GL261 model [53]. We observed that Lipo formulation had no significant cytotoxicity on GL261 cells (up to 160 µg/mL) or U87 cells (up to 80 µg/mL) and that t‐Lipo formulation had no significant cytotoxicity on GL261 cells (up to 160 µg/mL) or U87 cells (up to 40 µg/mL) (Figure S1a). These results were attributed to the biocompatibility of the developed formulations. In the hemolysis test, the formulation for the dose 40 µg/mL shows 0.39 ± 0.19% hemolysis, which is much lower than the 5% hemolysis rate limit for i.v. formulations required by the International Standards Organization (ISO, standard number 10993–4) [54]. This rate also confirmed the biocompatibility of the targeted liposomes with mouse blood (Figure S1b).

**FIGURE 2:**
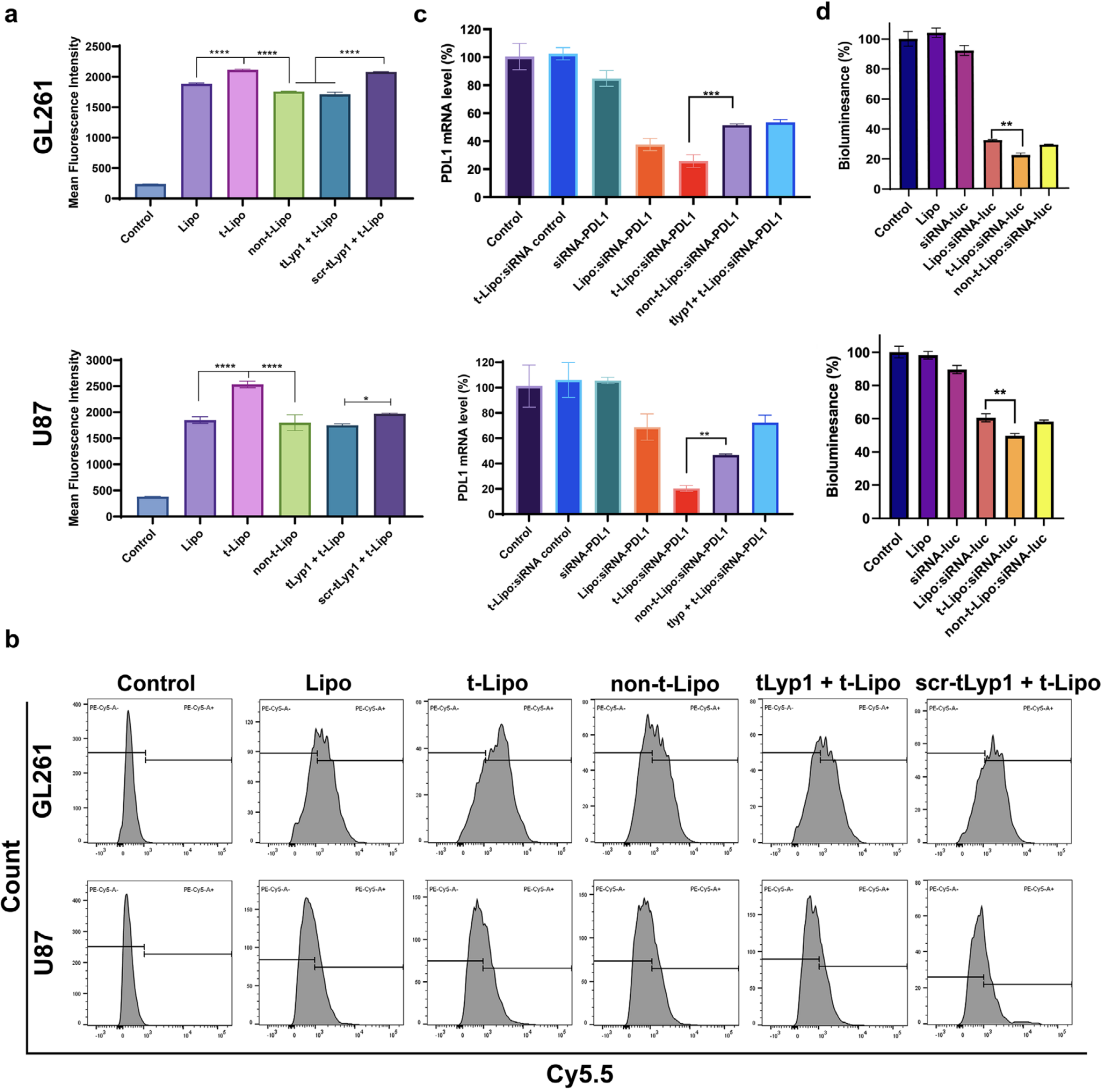
Analysis of targeted liposome uptake and gene silencing efficiency in glioma cells. (a) Bar graphs showing the mean fluorescence intensity of GL261 and U87 glioma cells were preincubated (or not) with free tLYP1 or scr‐tLYP1 (20 µM for competitive binding) for 30 min and treated with 40 µg/mL of either Control, Lipo, t‐Lipo, or non‐t‐Lipo, demonstrating the cellular uptake of Cy5.5‐labeled liposomes assessed by flow cytometry (n = 3). (b) Flow cytometry histograms illustrating the fluorescence intensity distribution of Cy5.5‐labeled liposomes in GL261 and U87 cells for the same groups. (c) Bar graphs depicting the relative PDL1 mRNA levels in GL261 and U87 cells treated with a similar experimental setup to (a) but using siRNA‐PDL1‐complexed liposomes, evaluated by qRT‐PCR to assess PDL1 knockdown efficiency (n = 3). (d) Bar graphs show the bioluminescence signal (as a percentage of control) in GL261 and U87 cells treated with siRNA‐luciferase‐complexed liposomes (n = 3).

The fluorescence dye Cy5.5 was conjugated to Lipo (without peptide) and targeted t‐Lipo formulations to evaluate their uptake in glioma cells by flow cytometry. The uptake of t‐Lipo was significantly higher in GL261 [55] or U87 [56] cells, both of which express NRP‐1 receptors, than that of the non‐t‐Lipo formulation (Figure 2a,b; ****P < 0.0001, t‐Lipo vs non‐t‐Lipo on GL261 and U87). Moreover, when the cells were preincubated with tLyp1 peptide, liposome uptake was significantly blocked (as compared to a scrambled tLYP1 control), suggesting that the observed uptake of t‐Lipo is tLyp1 peptide‐mediated (Figure 2a; ****P < 0.0001 for GL261 and *P = 0.018 for U87).

Next, the ability of the developed liposomes to deliver siRNAs to glioma cells was evaluated. Liposome variations carrying siRNA against PDL1 were applied to mouse GL261 and human U87 glioma cells. Their knockdown efficiency was analyzed using quantitative reverse transcription‐polymerase chain reaction (qRT‐PCR), and it was observed that preincubation with tLyp1 peptide reversed PDL1 knockdown (Figure 2c). At the 100 nM dose, t‐Lipo:siRNA‐PDL1 led to a 74 ± 4.6% decrease in PDL1 expression in GL261 cells (***P < 0.001, t‐Lipo:siRNA‐PDL1 vs. non‐t‐Lipo:siRNA‐PDL1). Similarly, the U87 glioma cell line treated with t‐Lipo:siRNA‐PDL1 showed a 79 ± 2.4% decrease for the same siRNA dose (**P < 0.01, t‐Lipo:siRNA‐PDL1 vs. non‐t‐Lipo:siRNA‐PDL1). Cells treated with Lipo:siRNA‐PDL1 (liposomes without peptide) or pre‐treated with 20 µM t‐Lyp1 for NRP‐1 blocking before receiving t‐Lipo:siRNA‐PDL1 showed a reduced gene‐silencing effect compared with the t‐Lipo:siRNA‐PDL1 group. The same liposome carrying a scrambled siRNA (siRNA‐control) showed no significant silencing effect (Figure 2c).

To validate these findings using an alternative siRNA, liposome variants complexed with siRNA‐luciferase (siRNA‐luc) were administered to luciferase‐expressing cells, and the subsequent luciferase activity was assessed. The cells treated with t‐Lipo:siRNA‐luc exhibited 22.3 ± 1.2% luciferase signal, whereas the non‐t‐Lipo:siRNA‐luc group demonstrated 29.3 ± 0.2% (**P = 0.0086) in GL261 cells. Similarly, the U87 glioma cell line treated with t‐Lipo:siRNA‐luc showed a bioluminescence of 49.6 ± 1.4% compared to 58.2 ± 1.0% for non‐t‐Lipo:siRNA‐luc (**P < 0.0070) (Figure 2d). These results indicate that our method successfully conjugates the tLyp1 peptide onto the Lipo surface, and that the resultant t‐Lipo exhibits high affinity and specificity for cells expressing the NRP‐1 receptor.

### 2.3 The Biodegradable Transducer Increases Permeability in an in vitro BBB‐on‐a‐Chip Model

**FIGURE 3:**
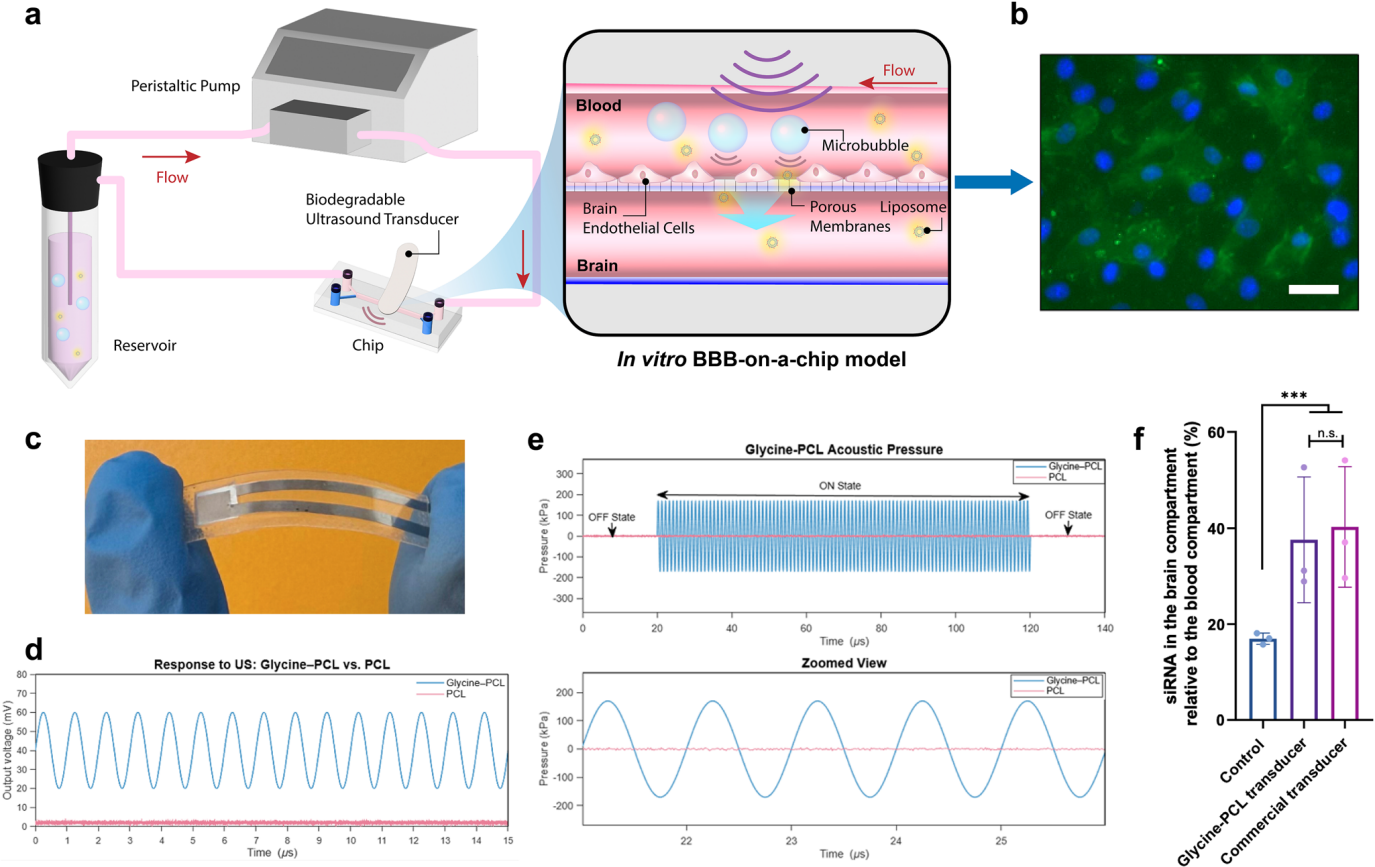
Overview of the in vitro BBB‐on‐a‐chip model and biodegradable US transducer characterization. (a) Schematic diagram of the in vitro BBB‐on‐a‐chip model setup, illustrating the use of a peristaltic pump, reservoir, and biodegradable US transducer to deliver microbubbles and liposomes across the blood‐brain barrier (BBB) to the brain compartment. (b) Fluorescence microscopy image of bEnd.3 cells in the in vitro BBB‐on‐a‐chip model, indicating high confluency. (Blue: Hoechst‐stained cell nuclei, green:  Alexa Fluor 488‐stained actin filaments of the cells) Scale bar = 50 µm. (c) Photograph of the fully biodegradable glycine‐PCL US transducer. (d) The open‐circuit voltage response to US for glycine‐PCL and PCL‐only films, demonstrating the piezoelectric properties of the glycine‐PCL transducer. (e) The acoustic pressure waveform generated by the glycine‐PCL transducer, showing the ON and OFF states with a zoomed view of the near‐sinusoidal pressure output. (f) The relative siRNA permeability in the brain compartment of the BBB‐on‐a‐chip model using glycine‐PCL transducer and commercial transducer, compared to the control group (n = 3).

In vitro 3D models of the BBB are increasingly recognized as valuable tools for predicting in vivo permeability before animal studies. These models enable the assessment of key BBB functions, including tight junction integrity and selective permeability [57]. Here, we utilized an in vitro BBB‐on‐a‐chip model to investigate the permeability of t‐Lipo:siRNA from the blood compartment to the brain compartment, with and without sonication (Figure 3a). The experiment was conducted on fully confluent bEnd.3 cells (Figure 3b).

The glycine‐PCL transducer was produced as mentioned in our recently published study [9], with its photograph presented (Figure 3c). In that previous publication, we have extensively characterized this biodegradable device for piezoelectric/US performance, and demonstrate (1) its biodegradability, (2) safety profile for brain implantation in rodents, and (3) a functional lifetime of at least 30 days (the time over which the device can still provide consistent US). Here, we re‐affirmed the key piezoelectric properties of glycine‐PCL nanofiber films and illustrated the device performance in Figure 3d,e. Representative output voltage waveforms generated by glycine‐PCL nanofiber films under 1 MHz US stimulations (see Methods for details) are shown (Figure 3d). The film was configured as a transducer by sandwiching it between two aluminum electrodes. Upon US stimulation, the mechanical stress induced on the glycine‐PCL film generated a distinct electrical output of ∼ 37 mV (blue trace) via the direct piezoelectric effect. In contrast, a PCL‐only film subjected to the same US conditions produced a negligible voltage response (< 5 mV; red trace). The same glycine‐PCL film can function as an US emitter, converting electrical energy into mechanical pressure via the converse piezoelectric effect (Figure 3e). The transducer with glycine‐PCL film was driven by a function generator to produce continuous US waves at 1 MHz (see Methods for details). The transducer with glycine‐PCL film generated distinct acoustic pressure, while the transducer with PCL‐only film produced only background noise. These results reconfirm that the glycine‐PCL film exhibits piezoelectric properties, demonstrating both direct and converse piezoelectric effects, whereas the PCL‐only film shows no such response. The negligible voltage and acoustic output generated by PCL‐only films confirm that ultrasound generation originates from the piezoelectric glycine‐PCL component rather than the implant structure itself. Accordingly, PCL‐only films were used as negative controls in our previous validation studies [9].

The in vitro BBB‐on‐a‐chip model was run after ensuring transducer functionality. t‐Lipo:siRNA complexes and fresh MBs were perfused to mimic blood flow. Then, a glycine‐PCL transducer or a commercial transducer with a 1 cm^2^ transducer head was placed on the parallel chips to receive US. After 5 min of sonication, the siRNA concentration in the lower compartment was quantified, and permeability (%) was determined based on the initial siRNA concentration in the circulating upper compartment. The control group without US showed 16.9 ± 1.2% permeability, compared to 37.5 ± 10.7% for the glycine‐PCL transducer and 40.2 ± 10.2% for the commercial transducer. These results indicate that our glycine‐PCL transducer increased permeability, which was not significantly different from that of the commercial transducer (P > 0.05, n.s) (Figure 3f). While increased permeability was observed in the in vitro BBB model, the absence of detectable tissue damage in vivo, together with prior validation of transient BBB opening [9], supports a controlled and non‐destructive mechanism of BBB modulation. Importantly, this approach also offers additional advantages, including biodegradability and the capacity for repeated applications, facilitating the efficient delivery of gene therapeutics across the BBB into deep brain tissues.

To further determine whether the increased permeability resulted from reversible barrier modulation rather than nonspecific endothelial injury, TEER recovery and endothelial viability were evaluated in blank bEnd.3 endothelial layers exposed to the device‐mediated ultrasound condition. TEER significantly decreased immediately after ultrasound exposure, indicating transient disruption of endothelial barrier integrity (Figure S2a). Importantly, TEER progressively recovered during the post‐sonication period and was not significantly different from the pre‐sonication baseline at later recovery time points. In parallel, endothelial viability, assessed using a CCK‐8 assay, remained comparable between the US (−) and US (+) groups (Figure S2b). These results support that the ultrasound condition used in this study enhanced permeability through transient and recoverable endothelial barrier modulation rather than persistent endothelial injury or overt cytotoxicity.

### 2.4 The Biodegradable Transducer Enhances Targeting Ability of t‐Lipo on GBM

Sonication‐mediated BBB opening is a promising technique for delivering nanoparticles and other therapeutic agents across the BBB to treat brain cancers [58]. A recent study has indicated that a novel mechanism called “cyclic jetting” facilitates MB‐mediated drug delivery by transiently opening the BBB. This process involves the use of US‐responsive MBs, which generate cyclic microjets when subjected to US waves. These microjets puncture cell membranes, enhance drug uptake, and allow therapeutic agents to cross the BBB [59]. Moreover, the ability of the BATUS platform to induce US‐mediated BBB opening has been previously validated in healthy brain models, where delivery of macromolecular tracers was observed only upon sonication, confirming active BBB modulation in the absence of disease‐related permeability [9]. Building on this established capability, the present study extends the platform by integrating US‐mediated BBB modulation with ligand‐directed targeting to further enhance delivery efficiency in a GBM model. In this context, we sought to leverage this combined mechanism to improve targeted gene delivery and thereby enhance therapeutic efficacy in the treatment of GBM.

**FIGURE 4:**
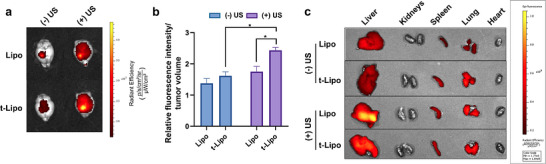
Enhanced brain targeting and biodistribution of targeted liposomes induced by transducer‐mediated BBB opening in a syngeneic, orthotopic GBM model in vivo. (a‐c) *Ex vivo* fluorescence imaging assessed the accumulation of Cy5.5‐labeled liposomes in the brain and internal organs 16 h after intravenous injection. (a) Representative *ex vivo* fluorescence images of dissected brains from mice bearing GL261 tumors, treated with either Lipo:Cy5.5 or t‐Lipo:Cy5.5, with or without prior sonication using the biodegradable glycine‐PCL transducer following microbubble administration. (b) Mean fluorescence intensity in the brains was quantified and normalized to tumor volume (n = 4, *P < 0.05). (c) Representative *ex vivo* fluorescence images of internal organs (liver, kidneys, spleen, lung, heart) from the same experiment illustrating biodistribution, with the top row indicating the organ locations analyzed.

One week after the tumor induction, tumor volume was monitored by bioluminescence imaging. The animals were then randomly divided into four groups (n = 4). Importantly, this biodistribution study was conducted prior to any therapeutic intervention and was designed solely to evaluate brain accumulation of fluorescently labelled formulations under different targeting and ultrasound conditions. The biodegradable transducer, with dimensions of 5×5 mm, was placed in the craniotomy region of the skull (Figure S3). Mice were administered an intravenous injection of either Lipo:Cy5.5 or t‐Lipo:Cy5.5 (40 µg in 100 µL PBS), or underwent sonication followed by the same formulations after 5 min. In the groups subjected to sonication, MBs were administered intravenously beforehand to enhance local cavitation of US pulses. This procedure temporarily disrupts the tight junctions between endothelial cells in the brain microvasculature, leading to an increase in BBB permeability. Sixteen hours post‐injection, the animals were euthanized, and their brains and internal organs were dissected and imaged for Cy5.5. The fluorescence intensity observed in the brain was markedly elevated following the administration of t‐Lipo:Cy5.5 in comparison to Lipo:Cy5.5 (*P < 0.05) (Figure 4a). Furthermore, mice subjected to sonication exhibited a significantly higher Cy5.5 signal in their brains relative to the non‐sonicated group (*P < 0.05) (Figure 4a,b), suggesting that the sonication by the transducer effectively enhanced delivery efficiency. While active targeting ligands such as tLyp1 are designed to enhance cellular uptake and tissue penetration, their efficiency is often hindered by the intact BBB [60]. In our study, we observed that the BATUS platform significantly amplifies the delivery of tLyp1‐conjugated liposomes compared to their systemic administration alone. This synergistic effect indicates that localized US‐mediated BBB opening is a critical requirement to fully realize the potential of targeted nanocarriers in GBM therapy, surpassing the limitations of ligand‐mediated transport across the BBB. It should be noted that increased accumulation in GBM may also be influenced by EPR‐like effects associated with tumor vascular leakiness [61]. However, the significantly higher signal observed in sonicated groups compared to non‐sonicated controls indicates that US‐mediated BBB modulation provides an additional contribution beyond baseline permeability.

Upon examination of the fluorescent signal within the internal organs, it was observed that Cy5.5 accumulation was most pronounced in the liver, followed by the spleen and lung. The Cy5.5 signal in the liver for the t‐Lipo:Cy5.5 group was marginally lower than that in the Lipo:Cy5.5 administered group (Figure 4c). Consequently, targeting may also play a role in mitigating potential liver toxicity associated with the formulation. The results indicate that the combination of targeting peptide conjugation and sonication by an ultrasonic transducer substantially improves the specificity of the formulation for the brain region. Because no therapeutic treatment was administered before biodistribution analysis, differences in fluorescence signal are not attributable to treatment‐induced changes in tumour burden.

### 2.5 BATUS Induced Antitumoral Activity of t‐Lipo:siRNA‐PDL1 in a Syngeneic Orthotopic GBM Model and Showed High Safety Profile in Small and Large Animals

**FIGURE 5:**
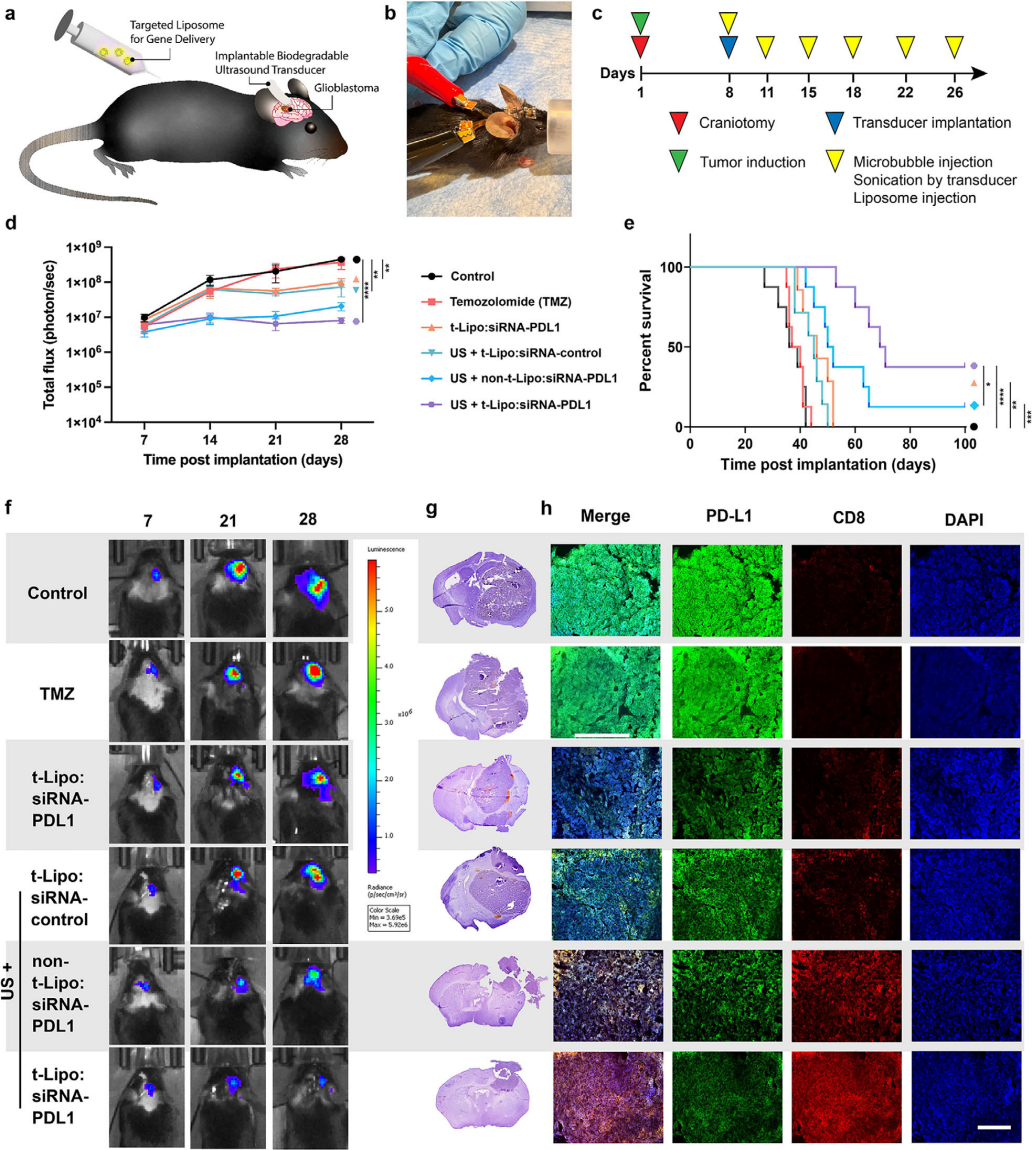
Therapeutic efficacy and survival benefits of the BATUS platform in the treatment of orthotopic GMB tumors in mice. (a‐c) Experimental design and timeline for BATUS‐mediated treatment of C57BL/6 mice bearing GL261 tumors. (a) Schematic of our implanted biodegradable US transducer to repeatedly deliver newly formulated tumor‐targeting liposomes through the BBB for brain cancer gene therapy. (b) Intraoperative image showing the sonication procedure. (c) Experimental timeline: craniotomy and tumor induction (day 1), transducer implantation (day 8), microbubble injection, sonication, and liposome administration (days 8, 11, 15, 18, 22, and 26). (d) Tumor growth monitored by total photon flux via bioluminescence imaging over time post‐implantation (days 7, 21, and 28) for control, TMZ, t‐Lipo:siRNA‐PDL1, US + t‐Lipo:siRNA‐control, US + non‐t‐Lipo:siRNA‐PDL1, and US + t‐Lipo:siRNA‐PDL1 groups (n = 8, **P < 0.01, ****P < 0.0001 vs. control),  the data are presented as the mean ± standard error of the mean (SEM). Terminal tumour‐burden comparisons were further supported by longitudinal analyses of the bioluminescence data, including a linear mixed‐effects model (REML; treatment × time interaction, P < 0.001) and area‐under‐the‐curve analysis (AUC; days 7–21, one‐way ANOVA, P < 0.001). (e) Kaplan‐Meier survival curves showing percent survival overtime post‐implantation (days 0–100) (****P < 0.0001 control vs. US + t‐Lipo:siRNA‐PDL1, *P = 0.0468 US + non‐t‐Lipo:siRNA‐PDL1 vs. US + t‐Lipo:siRNA‐PDL1). (n = 8) (f) Bioluminescence images from a mouse in each group are shown over time. (g) Representative H&E‐stained brain sections on day 28 post‐implantation showing tumor morphology across treatment groups. (h) Immunohistochemical analysis of brain sections stained for PD‐L1 (green), CD8 (red), and DAPI (blue), with merged images indicating PD‐L1 expression and CD8 + T cell infiltration. Scale bar = 200 µm.

Finally, we evaluated the ability of the BATUS technology to deliver siRNAs for targeted anti‐PD‐L1 therapy to GBM in a syngeneic, orthotopic GL261 GBM mouse model. The depiction and timeline of the experiment are described (Figure 5a–c; FigureS3). Briefly, at the beginning (day 0), a craniotomy was performed, and tumor cells were inoculated into the animal brains. On day 8, the biodegradable US transducer was implanted and positioned on the craniotomy defect. A bolus of 30 µl of MBs (2×10 ^8^ MBs/mL) was injected into the animal through the retro‐orbital route. Following US treatment, t‐Lipo:siRNA‐PDL1 was administered to the animals via intravenous injection (Figure 5a). This procedure was repeated for a total of six sessions. This approach highlights the advantage of our implanted US transducers: they provide highly localized stimulus delivery and eliminate the attenuation and positioning hurdles of external US methods. Control and sham groups included mice receiving only PBS (negative control), temozolomide (TMZ), t‐Lipo:siRNA‐PDL1 without US treatment, non‐t‐Lipo:siRNA‐PDL1, or t‐Lipo:siRNA‐control following US sonication. Tumor growth was monitored by Fluc bioluminescence imaging over time, and mice were monitored for survival analysis (Figure 5d–f).

TMZ did not have a significant effect on tumor growth or mouse survival as compared to the control group, with median survival out to 37.5 and 38.5 days, respectively (Figure 5d,e). While TMZ remains a traditional treatment for GBM, it provides limited efficacy in certain models^23^. In the literature, TMZ treatment interestingly showed a negative effect on the number of intratumoral CD4^+^ and CD8^+^ T cells as compared to mock, highlighting the necessity of novel approaches^24^. On the other hand, t‐Lipo:siRNA‐PD‐L1 without sonication produced a moderate but significant antitumor effect compared with the control group (Figure 5d,f). By day 28, the bioluminescence total flux in the t‐Lipo:siRNA‐PDL1 group was markedly lower than that of the control (9.9 ± 0.7 × 10^7^ vs. 4.5 ± 0.4 × 10^8^, respectively; **P < 0.01). This therapeutic effect was further supported by a significant extension in median survival, increasing from 37.5 days in the control group to 46 days in the t‐Lipo:siRNA‐PDL1 (Figure 5e; P = 0.0048), confirming the efficacy of the targeted siRNA delivery system. Similarly, sonication combined with targeted Lipo:siRNA control (t‐Lipo:siRNA‐control) led to a significant reduction in tumor growth compared with the control group (Figure 5d,f). At day 28, total flux was markedly lower in the sonication + t‐Lipo:siRNA‐control group than in controls (7.2 ± 0.7 × 10^7^ vs. 4.5 ± 0.4 × 10^8^, respectively; **P < 0.01). This was accompanied by a significant improvement in median survival (45 days vs. 37.5 days in controls; **P = 0.0078). Previous studies suggest that sonication itself can induce immunogenic cell death, promoting the release of tumor‐associated antigens and triggering antitumor immune responses [64, 65]. Notably, the combination of sonication and targeted t‐Lipo:siRNA‐PDL1 produced the most robust therapeutic outcome. Tumor growth was profoundly suppressed (day 28 total flux: 8.1 ± 0.4 × 10^6^, ****P < 0.0001 vs. control) and a marked extension in median survival (70 days; ****P < 0.0001 vs. control). In addition, survival in this group was significantly improved compared with the US sonication + non‐t‐Lipo:siRNA‐PDL1 group (51 days, *P = 0.0468 vs US + t‐Lipo:siRNA‐PDL1) (Figure 5d–f). To further assess tumour progression over time, longitudinal bioluminescence data were analyzed using a linear mixed‐effects model (REML). This analysis revealed a significant treatment × time interaction (P < 0.001) and a significant main effect of treatment (P < 0.0001), indicating that tumour‐growth trajectories differed among treatment groups throughout the study. Consistent with these findings, cumulative tumour burden, quantified as the area under the log10‐flux curve (AUC; days 7–21), also differed significantly among groups (one‐way ANOVA, P < 0.001).This demonstrates that the combined approach yields a highly synergistic therapeutic effect, whereas simpler, non‐targeted or non‐US‐assisted methods fall short. Importantly, these results enable a direct comparison of the individual and combined contributions of targeting and US‐mediated BBB modulation. While targeted liposomes alone (t‐Lipo:siRNA‐PDL1) produced a moderate therapeutic effect, and US combined with non‐targeted formulations also resulted in partial tumor suppression, the combination of US and targeted liposomes yielded a markedly superior outcome in both tumor reduction and survival. Although the current therapeutic study did not include a matched non‐piezoelectric implant control, device characterization presented here and prior validation studies demonstrated that PCL‐only implants do not generate meaningful acoustic output or BBB modulation. These results indicate that neither BBB opening nor cellular targeting alone is sufficient to achieve the full therapeutic benefit, and that the BATUS platform derives its efficacy from the synergistic integration of these two mechanisms.

These findings were corroborated by *ex vivo* histological analysis utilizing hematoxylin and eosin (H&E) staining (Figure 5g). Although the current therapeutic study did not include a matched non‐piezoelectric implant control, device characterization presented here and prior validation studies demonstrated that PCL‐only implants do not generate meaningful acoustic output or BBB modulation. Furthermore, immunohistochemical staining of brain sections revealed that the combination of US and t‐Lipo:siRNA‐PDL1 resulted in a reduction of PD‐L1 expression while enhancing the recruitment of CD8^+^ T cells (Figure 5h), consistent with the tumor growth and survival analysis presented above. To strengthen this histological assessment, PD‐L1 and CD8 fluorescence signals were further quantified from the tumor sections (n = 5). Quantitative image analysis showed reduced PD‐L1 signal in the treated groups, with the lowest PD‐L1 expression observed in the US + t‐Lipo:siRNA‐PDL1 group (Figure S5). In contrast, CD8 signal was increased following treatment, with the highest CD8+ T‐cell signal detected in the US + t‐Lipo:siRNA‐PDL1 group. These quantitative staining results support the representative immunohistochemical images and are consistent with the observed suppression of tumor growth and prolonged survival in this treatment group. Collectively, these data suggest that BATUS technology effectively enhances liposome‐based siRNA delivery for targeted anti‐PD‐L1 therapy, thereby contributing to reduced tumor growth and improved survival in mice. While these findings highlight the potential of the BATUS platform, established GBM models may not fully recapitulate the complexity of human GBM, warranting further evaluation in clinically relevant systems.

One of the most critical criteria to be evaluated prior to clinical translation of the implanted US transducer and the BATUS platform is safety. While the long‐term safety of the biodegradable implantable transducer has previously been shown in mice for up to 6 months [9], here we re‐evaluated and re‐affirmed its local and systemic safety in a large‐animal brain model and overall safety of the BATUS platform in subcutaneous mouse model (Figure S4). More specifically, in a porcine short‐term study, our biodegradable device was positioned over the dura. The lateral flaps of the device were affixed onto the surrounding bone for a two‐week implantation period. At the end of this period, the pigs were euthanized, and tissue samples were examined histologically. H&E staining of local brain tissue (Figure S6) in contact with the implant showed a lack of host immune and cellular infiltration responses, compared to the control group without transducer implantation. Furthermore, Masson's trichrome staining of the same tissue field revealed a lack of host foreign body response and collagen deposition. Locally, both H&E and Masson's trichrome staining also appeared no different from the contralateral control tissue (Figure S7a). In addition to examining the local response, to assess possible systemic effects following implantation, H&E and Masson's trichrome staining were also carried out on the spleen, liver, and kidney from the same animals. These organs were selected for analysis as a consequence of being involved in filtration of the circulatory system of the body, as well as also being positioned at various distances from the implant location. None of the tissues were different from the baseline tissue from the control craniotomy‐alone pig (Figure S7b). Although the implantation of the BATUS transducer requires an initial surgical procedure, its risk‐benefit profile is highly favorable for the treatment of aggressive brain malignancies such as GBM. Compared to transcutaneous systems such as SonoCloud, the fully integrated, biodegradable design of our device enables repeated, localized, and on‐demand acoustic targeting without the need for repeated transcutaneous energy transmission or systemic toxicity [7, 9]. The safety of the BATUS platform in a syngeneic, orthotopic GBM mouse model was also evaluated. No abnormal behavior was observed after craniotomy or during the treatment period (Movie S1). The safety of the targeted liposome treatment, together with sonication, was also assessed through body weight during this mouse study, and histological analysis was performed after the last dose of the treatment. No histological changes were observed in the internal organs or in the skin compared with those in the control group receiving PBS without biodegradable transducer implantation (Figures S7 and S4a,b). While the present study demonstrates effective delivery, target engagement, therapeutic efficacy, and safety of the BATUS platform in a GBM model, future in vivo pharmacokinetic analyses could further strengthen its translational potential. Taken together, these findings indicate that BATUS technology could offer a safe and effective platform for addressing challenging brain tumors.

## 3 Conclusion

Here, we present BATUS as a biodegradable acoustic targeting strategy that enables repeated and localized BBB modulation for efficient gene delivery. By integrating an implantable US transducer with peptide‐targeted liposomal carriers, this system overcomes key biological barriers and achieves effective gene silencing and therapeutic outcomes in orthotopic GBM models. Importantly, BATUS demonstrates a favorable safety profile in both small and large animal studies, supporting its translational potential.

While these findings highlight the strong potential of BATUS, several aspects require further investigation for clinical translation. Broader validation with diverse genetic cargos, including siRNA, mRNA, CRISPR components, and antisense oligonucleotides, as well as combination therapies, will be essential to fully exploit the platform. In addition, the safety evaluation in pigs remains preliminary and warrants longer‐term studies with larger cohorts. In particular, detailed in vivo pharmacokinetic and biodistribution analyses in big animal models are needed to determine the circulation behavior, brain accumulation, systemic distribution, clearance, and dose/time relationships of the liposomal siRNA formulation following repeated ultrasound‐assisted delivery. Further characterization of the biodegradable transducer will also be important, including analysis of degradation products, metabolic fate, and tissue responses over longer implantation periods. The GBM model used in this study, while suitable for demonstrating therapeutic efficacy, may not fully reflect the clinical heterogeneity of human disease, underscoring the need for validation in more representative models. Furthermore, adaptation of the implantable US system to human anatomy and optimization of treatment parameters will be necessary.

Collectively, the results presented here position BATUS as a robust and adaptable gene delivery platform, offering a safe and effective strategy for repeated and targeted delivery of therapeutic nucleic acids to the brain. This approach provides a strong foundation for advancing gene‐based therapies in GBM and may enable future applications in other challenging brain diseases.

## 4 Experimental Section

### 4.1 Materials

DOTAP and DOPE were purchased from Cayman Chemical Co. (MI, USA). DSPE‐PEG(2000)‐DBCO was obtained from Avanti Polar Lipids (AL, USA). Custom‐designed tLyp1 and scrambled tLyp1 (scrtLyp1) peptides with azide groups were synthesized by GenScript Biotech (NJ, USA). Unless otherwise specified, materials and reagents were obtained from Fisher Scientific or Sigma‐Aldrich.

For the preparation of fluorescently labeled liposome, sulfo‐cyanine 5.5 azide was obtained from Lumiprobe Co. (MD, USA). Including siRNA specific for firefly luciferase (code:AM4629) and control siRNA (code: AM4613), all siRNAs were purchased from Invitrogen (CA, USA). siRNA sequences for PDL1 siRNA were as follows for sense and antisense strands: human‐specific siRNA‐PDL1:5′‐GCAUAGUAGCUACAGACAGtt‐3′ and 5′‐CUGUCUGUAGCUACUAUGCtg‐3′; mouse‐specific siRNA‐PDL1:5′GCGAAUCACGCUGAAAGUCtt‐3′ and 5′‐ GACUUUCAGCGUGAUUCGCtt‐3.’ The primer pairs used for gene detection were obtained from Eurofins, Germany. The specific primer sequences are as follows: Mouse β‐actin, 5′‐GTGACGTTGACATCCGTAAAGA‐3′ (forward) and 5′‐GCCGGACTCATCGTACTCC‐3′ (reverse); Human β‐actin, 5′ CATGTACGTTGCTATCCAGGC‐3′ (forward) and 5′‐CTCCTTAATGTCACGCACGAT‐3′ (reverse); Mouse PDL1, 5′‐GCTCCAAAGGACTTGTACGTG‐3′ and 5′‐TGATCTGAAGGGCAGCATTTC‐3′ (reverse); Human PDL1, 5′‐TGGCATTTGCTGAACGCATTT‐3′ and 5′‐TGCAGCCAGGTCTAATTGTTT T‐3′ (reverse).

### 4.2 Cell Culture

Human GBM cell line expressing luciferase (U87) and mouse brain endothelial cells (bEnd.3) used in in vitro BBB‐on‐a‐chip model were purchased from the American Type Culture Collection (ATCC). The mouse GBM cell line expressing luciferase (GL261) was purchased from Creative Biolabs, NY, USA. U87 and GL261 GBM cell lines express integrin NRP‐1 receptor [56, 66, 67]. The cells were maintained in DMEM, GlutaMAX medium with 10% FBS, and 1% penicillin/streptomycin. All cell lines were confirmed to be mycoplasma‐free and were maintained in a humidified environment at 37°C with 5% CO_2_.

### 4.3 Preparation of the Delivery Systems

The liposome (Lipo) formulation was prepared using an adapted film hydration protocol. For this purpose, DOTAP, DOPE, and cholesterol were dissolved in chloroform in a molar ratio of 1:0.1:1. DSPE‐PEG(2000)‐DBCO was then added to this solution at a weight ratio of 29:1 (total lipid:DSPE‐PEG(2000)‐DBCO, w/w). The solution underwent evaporation for 30 min at a temperature of 45°C. The resulting thin‐film layer was then rehydrated with ultrapure H_2_O and subjected to sonication for 15 min using a probe‐type sonicator (Branson SFX250 Sonifier; CT, USA). Ultimately, the formulation achieved a lipid concentration of 2 mg/mL. At this stage, the Lipo formulation containing DBCO was synthesized and is subsequently ready for linkage with commercially produced azide‐containing peptides through copper‐free click chemistry [53]. The tLyp1/scrtLyp1 peptide was subsequently conjugated to the reactive DBCO groups of Lipo at a 1:1 M ratio of peptide to Lipo at a final concentration of 22 µM to obtain t‐Lipo or non‐t‐Lipo, respectively. The mixture was incubated in a rotating mixer for 2h. Any unconjugated tLYP1/ scrtLYP1 was removed by three washing cycles using 10 kDa ultrafiltration tubes (Millipore, USA).

### 4.4 Preparation of t‐Lipo:siRNA Complexes

To identify the optimum complexation protocol, various P^−^/N^+^ ratios of siRNA to liposome (P^−^/N^+^, 1:1, 1:2, 1:3, 1:4, and 1:5) were tested by incubating them on a benchtop shaker for 30 min at room temperature, allowing the siRNA to bind to the liposomes through electrostatic interactions. The efficiency of siRNA complexation was evaluated using a gel retardation assay with a 2% agarose gel, as previously described [28].

### 4.5 Characterization of the Delivery Systems

The particle size, polydispersity index (PDI), and surface charge of the liposomes [Lipo, t‐Lipo, and t‐Lipo:siRNA] were assessed using dynamic light scattering (NanoZS, Malvern). Three µL of the liposome solutions at a concentration of 0.5 mg/mL were placed onto carbon‐coated copper grids. After being adsorbed for two min, the samples were rinsed with 100 µL of a 0.5% uranyl acetate aqueous solution. Excess uranyl acetate was removed with filter paper, and the grids were allowed to air dry. Visualization of the grids was performed using an FEI Tecnai Biotwin TEM (FEI, Eindhoven, the Netherlands) operating at 80 kV.

The physicochemical stability of the Lipo and t‐Lipo, over 180 days at a temperature of 4°C was evaluated by measuring their size, PDI, and zeta potential on days 0, 15, 30, 60, 90, and 180 to detect any changes. In all experiments, liposome:siRNA complexes were freshly prepared immediately before use.

### 4.6 In vitro siRNA Release Study and Evaluation of Entrapment Efficiency

The release of siRNA from the t‐Lipo:siRNA complex (1:2, P^−^/N^+^) was assessed over a period of 168h. A 200 µL sample was immersed in 4 mL of PBS, placed into a dialysis bag (Pure‐A‐Lyzer Mini12000, Sigma, USA), and then incubated in a thermal shaker set to 100 rpm at 37°C. At each time point, the release buffer was completely replaced with fresh PBS. The RiboGreen Assay (Quant‐it RiboGreen RNA Assay Kit, Thermo Fisher Scientific, USA) was employed to quantify the amount of siRNA released into the buffer.

siRNA was associated with cationic liposomes primarily via electrostatic interactions at the lipid surface. In this study, entrapment efficiency is used in an operational sense to denote the total fraction of siRNA associated with the liposomal formulation [68]. To assess the efficiency of siRNA entrapment, a 1% (w/v) solution of Triton X‐100 was employed as a detergent to break down the liposome structure and release the siRNA. The RiboGreen Assay is used to measure the total siRNA in samples, both with and without Triton‐X treatment. To determine the amount of siRNA that is entrapped, the free siRNA is subtracted from the total siRNA. The entrapment efficiency (EE) was calculated by dividing the free siRNA by the total siRNA present in the sample, as shown in the equation below:

$$EE\%=\left(\frac{total\;siRNA-free\;siRNA}{total\;siRNA}x100\right)$$

### 4.7 Evaluation of Cytotoxicity and Hemocompatibility

The in vitro cytotoxicity of Lipo and t‐Lipo formulations was investigated on U87 and GL261 cell lines. Cells (5 × 10^3^ cells/well) were plated in 96‐well plates and exposed to varying concentrations of the liposomes (up to 160 µg/mL based on lipid components) for 24 h. The proportion of viable cells was assessed using the Alamar Blue cell viability assay (Thermo Fisher Scientific, USA). Cell viability was determined by normalizing the fluorescence of the media from treated cells to that of untreated cells.

To determine the effect of the t‐Lipo:siRNA complex on erythrocytes, hemocompatibility studies were performed [69]. Blood samples from C57BL/6 mice were collected in EDTA‐containing microcentrifuge tubes. Blood cells and plasma were separated by centrifugation at 5000 rpm for 10 min. The erythrocytes from the pellets were rinsed and then re‐suspended in PBS, and subjected to hemolysis assay. Erythrocytes (200 µL) treated with PBS (800 µL) were utilized as the negative control, while erythrocytes (200 µL) treated with 2% Triton X‐100 (800 µL) served as the positive control. Both the positive and negative control samples, along with the test samples comprising erythrocytes (200 µL) treated with 800 µL of test samples (t‐Lipo:siRNA, at concentrations of 320, 40, or 20 µg/mL in terms of lipid compounds, in PBS), were placed in 2 mL microcentrifuge tubes and incubated at 37°C for 2h in an incubator shaker. Subsequently, the microtubes were opened and allowed to stand for 10 min to facilitate hemoglobin oxidation. A volume of 20 µL of the oxidized samples from each tube was then transferred in triplicate to a 96‐well microplate. Subsequently, 180 µL of PBS was introduced into each well, and the plate was shaken for 5 min. The absorbance of all samples was then recorded at 540 nm using a microplate reader. The percentage of hemolysis was determined using the following equation.

$$\begin{array}{c} \%\mathrm{Hemolysis}=\frac{\begin{array}{c} Absorbance\;\left(sample\right)\\ \;-Absorbance\;\left(negative\;control\right) \end{array}}{\begin{array}{c} Absorbance\;\left(positive\;control\right)\\ \;-Absorbance\;\left(negative\;control\right) \end{array}}x100 \end{array}$$

### 4.8 Analysis of in Vitro Targeting Ability

To assess the cellular uptake of liposomes, sulfo‐cyanine 5.5 azide (Cy5.5) was conjugated to the liposomes via a DBCO strand utilizing click chemistry. Unbound Cy5.5 was eliminated using ultracentrifugal filters (10 kDa MWCO, Amicon). To investigate the targeting efficacy in vitro, 40 µg/mL (based on lipid components) of Cy5.5‐labeled Lipo, t‐Lipo, or non‐t‐Lipo was administered to GL261 or U87 cells at 37°C for 3h. In the blocking assays, cells were pre‐incubated with tLyp1 or scr‐tLyp1 peptide at a concentration of 20 µM for 30 min before the addition of liposomes.

Subsequently, the cells were collected using trypsin and washed three times with PBS. Flow cytometry (LSR Fortessa, BD Biosciences, CA, USA) was then employed to measure the fluorescence intensity of 10,000 cells from each sample. FlowJo software V10 (TreeStar, Ashland, DE, USA) was used for data analysis. The proportion of cells positive for Cy5.5 was assessed by comparing it with the PBS‐treated control group.

### 4.9 Evaluation of the Silencing Efficiency

Human U87 or mouse GL261 cells were seeded in 12‐well plates at 5×10^4^ cells/well and treated with formulations of Lipo, t‐Lipo, or non‐t‐Lipo carrying siRNA‐PDL1 (100 nM, concerning siRNA) specific to humans or mice, respectively. To verify the targeting capability of t‐Lipo, which relies on tLyp1, one set of cells was pre‐treated with a 20 µM concentration of tLyp1 peptide for 30 min prior to the addition of liposomes. Scrambled siRNA (siRNA control) was used as a negative control. Following a 24h‐period, the cells were rinsed with PBS and then placed in a growth medium for another 24h. Subsequently, the cells were harvested, and RNA was extracted and examined using qRT‐PCR with primer pairs specifically targeting PDL1, while β‐actin served as the control gene. The results were assessed using the ^ΔΔ^CT method, with untreated cells used as the reference/mock. Each experiment was repeated in triplicate at least three times.

Both U87 and GL261 cells were purchased as expressing firefly luciferase as mentioned in the method section. In a white 96‐well plate, the cells were seeded at a density of 5 × 10^3^ cells per well in triplicate 1 day before treatment. Lipo, t‐Lipo, or non‐t‐Lipo carrying siRNA‐luciferase (100 nM, concerning siRNA) was added to cells with fresh media, and incubation was continued for 48h. After removing the medium, the luciferase expression in the cells was assessed using the Steady‐Glo Luciferase Assay System (Promega, WI, USA) with a BioTek Synergy HTX Multi‐Mode Microplate Reader (Agilent Technologies, CA, USA). Each experiment was repeated in triplicate and at least three times.

### 4.10 In Vitro BBB‐on‐a Chip Model

The Be‐Doubleflow Standard microfluidic chip (BeonChip, Zaragoza, Spain), which consists of two parallel microchannels separated by a porous polyethylene terephthalate (PET) membrane (pore diameter 0.4 µm, porosity 10%) were used. bEnd.3 cells were trypsinized, counted with a hemocytometer, resuspended to a final concentration of 5 × 10^6^ cells/mL in the growth medium, seeded in the upper channel, and incubated at 37°C in a humidified incubator with 5% CO_2_ until reaching 100% confluency. Confluency of the bEnd.3 cells in the in vitro BBB‐on‐a‐chip model was confirmed by fluorescence microscopy after staining nuclei with Hoechst and actin filaments with Alexa Fluor 488. The cell medium was replaced with fresh medium every other day. On the third day of cell seeding on the microfluidic device, the upper channel of the BBB Chips was attached to a peristaltic pump (Masterflex Ismatec Reglo Independent Channel Control (ICC) Peristaltic Pumps, Avantor, PA, USA), and fresh medium containing MBs (at a concentration of 2 × 10^8^ bubbles/mL) and t‐Lipo:siRNA complexes (40 µg/mL) were perfused at a flow rate of 400 µL/min to mimic blood flow and allowed to run for 5 min. Then, a glycine‐PCL transducer (10 mm × 10 mm) or commercial transducers with a 1 cm^2^ transducer head were placed on the parallel chips and set to operate at 1.5 MHz, with a burst rate of 10 Hz and a duty cycle of 20%, using the same input voltage for two 30‐s shots, each separated by a 30‐s interval. Five min after sonication, 50 µL of sample was taken from the lower compartment, and the RiboGreen Assay (Quant‐it RiboGreen RNA Assay Kit, Thermo Fisher Scientific, USA) was employed to measure the amount of siRNA in the media. A group without sonication was also included in this study. Triton X‐100 1% (w/v) was employed as a detergent to break down the liposome structure and release the siRNA before RiboGreen Assay. The permeability (%) of siRNA was calculated as the ratio of siRNA in the lower compartment to the total siRNA in the circulating media. Each experiment was repeated in triplicate.

### 4.11 Preparation of the Biodegradable US Transducer

The biodegradable glycine‐based transducer made of a stable, highly piezoelectric nanofiber film of glycine crystals embedded in a polycaprolactone polymeric matrix was produced as mentioned in our recently published study [9]. To obtain glycine crystals, glycine powder was dissolved in UPH_2_O, and the solutions were dropped onto clean glass slides. The droplets were then left to dry under ambient conditions. β‐Glycine crystals formed via slow evaporation to ensure the formation of the piezoelectric β‐phase. The crystals were carefully removed from the glass slides and ground in a microtube homogenizer using metal beads during 2 min. Then, ground crystals were added to the PCL solution (20 wt% PCL in a mixture of dichloromethane (DCM) and N,N‐dimethylformamide (DMF)) and vigorously stirred overnight at a final glycine:PCL ratio of 1:1 (w/w) to ensure uniform dispersion. Electrospinning was conducted under ambient conditions (30 ± 10% humidity and 22°C). The precursor solution was loaded into a 5 mL syringe fitted with a 21‐gauge flat‐tip stainless‐steel needle. The solution was delivered at a rate of 2 mL/h using a programmable syringe pump (New Era Pump Systems, NY, USA). A 14 kV voltage was applied to the needle, and fibers were collected on a grounded aluminum drum (15 cm diameter) rotating at 4000 rpm [70]. The needle‐to‐collector distance was maintained at 8.5 cm. After spinning, the mats were dried under vacuum at 25°C for 72 h to eliminate the residual solvents.

Electrospun mats were cut into 5 mm × 5 mm films. PLA encapsulating films were produced by compression molding at 200°C followed by quenching using dry ice.

Two 5 mm × 5 mm squares attached to 1‐mm‐wide wires were excised from a 25‐µm‐thick sheet of Mo (ESPI Metals). The electrodes were then hot‐embossed into PLA at 110–130°C using CarverPress (IN, USA). Subsequently, the films were sandwiched between the two electrodes that were embedded in PLA, and the resistance was verified using a multimeter (Extech, MN35, NH, USA)). All the edges of the encapsulator were then sealed three times using a sealing machine (ULINE, USA) for 5s. The completed transducers were stored in a vacuum desiccator at room temperature. Prior to biological applications, the devices were sterilized by immersion in 70% ethanol for ∼30 min, followed by exposure to UV light for another 30 min.

### 4.12 Characterization of Biodegradable Piezoelectric Transducer

We performed open‐circuit voltage measurements under US and US transmission tests, based on the direct and converse piezoelectric effects respectively, to confirm that the piezoelectricity of the biodegradable glycine‐PCL transducer originates from the glycine crystals rather than the PCL matrix. For both tests, a PCL nanofilm without glycine crystals was used as a negative control.

For measurement of open‐circuit voltage under US, the transducers were subjected to ultrasonic waves at 1 MHz and 0.5 Wcm^−2^ by a SoundCare Plus commercial US system (Sonicare Medical Inc.). The open‐circuit voltage from the transducer was then monitored using an oscilloscope (PicoScope 4824).

For the US transmission test, a calibrated needle hydrophone (SN‐3329, Precision Acoustics) with sensitivity 0.2 mVPa^−^^1^ and the transducers were mounted and immersed in a DI water tank. The hydrophone was positioned axially 2 cm away from the transducer surface and aligned with the transducer to maximize the signal reception. A continuous sinusoidal wave at 1 MHz with an amplitude of 0.1 *V_rms_*, generated by a function generator (BK Precision, 4054B), was amplified by a radiofrequency (RF) power amplifier (E&I RF Amplifier 1040 L) and applied to the transducer.

The acoustic pressure values were derived from the hydrophone's voltage output based on the calibrated sensitivity of the hydrophone.

### 4.13 TEER Recovery and Endothelial Viability After Ultrasound Exposure

To assess endothelial barrier integrity and recovery after device‐mediated ultrasound exposure, blank bEnd.3 endothelial layers were prepared under the same culture conditions used for the in vitro BBB‐on‐a‐chip study. TEER was measured using an EVOM Manual Meter for TEER measurement (World Precision Instruments, FL, USA) before ultrasound exposure and at defined time points after sonication. Briefly, resistance values were measured at −0.25, 0, 0.5, 1, 2, 4, and 24 h relative to ultrasound exposure. Background resistance from blank inserts/chips without cells was subtracted, and TEER values were calculated by multiplying the corrected resistance by the membrane surface area and reported as Ω·cm^2^.

Endothelial cell viability after ultrasound exposure was evaluated using a CCK‐8 assay according to the manufacturer's protocol. Blank bEnd.3 endothelial layers were assigned to US (−) or US (+) groups. After ultrasound exposure, CCK‐8 reagent was added to the cells and incubated for 1 h at 37°C. Absorbance was measured at 450 nm using a microplate reader. Cell viability was calculated by normalizing absorbance values to the US (−) control group.

### 4.14 In Vivo GBM Model

All small animal studies were approved by the University of Connecticut Institutional Animal Care and Use Committee (IACUC) (protocol number A24‐006) and adhered to the guidelines established by the National Institutes of Health Guide for the Care and Use of Laboratory Animals. A syngeneic, orthotopic GBM model was developed using the GL261 cell line in 8−10‐week‐old C57BL/6 female mice. The GL261 cell line was selected for its role as a model for GBM immunotherapy research. It does not necessitate an immunocompromised environment, and it can closely simulate the growth patterns and immune responses characteristic of human GBM [71, 72].

Mice were anesthetized with 3% isoflurane in oxygen during the procedure. An incision was created along the midline of the skull, and a craniotomy defect with a diameter of 3 mm was established at the specified coordinates: 2.5 mm lateral, 0.5 mm anterior to bregma. Then, 100.000 or 250.000 GL261 cells with luciferase expression were implanted stereotactically in 2 µL of PBS using a 30‐gauge Hamilton syringe, reaching a depth of 2.5 mm from the skull's surface.

To track tumor development, bioluminescence imaging was conducted using a Xenogen IVIS 200 imaging system (PerkinElmer, Waltham, MA, USA). In summary, mice received an intraperitoneal injection of D‐luciferin at a dose of 150 µg/g of body weight and were then placed in the imaging chamber. Fifteen min following the injection of luciferin, imaging was conducted, and the intensity of the signal was assessed using Living Image software (version 3.0; Xenogen Imaging Technologies, Waltham, MA, USA).

### 4.15 Transducer Implantation and Sonication

Following tumor development, a biodegradable transducer made from glycine, measuring 5×5 mm, was placed in the craniotomy area of the skull. After closing the incision with surgical clips, the mice were given a 10 min rest period. Before the sonication procedure, a 30 µL bolus of US contrast agent (VisualSonics, VS‐11694) containing MBs at a concentration of 2 × 10^8^ bubbles/mL was administered to the animal via the retro‐orbital route. The transducer was set to operate at 1.5 MHz, with a burst rate of 10 Hz and a duty cycle of 20%, using the same input voltage for two 30‐s shots, each separated by a 30‐s interval [9].

### 4.16 Investigation of BBB Distribution and Targeting

To examine the impact of sonication on liposome uptake by GBM, one week after the implantation of 25 × 10^4^ GL261‐Fluc cells in C57BL/6 mice, each group (n = 4) of mice received an intravenous injection of either Lipo:Cy5.5 or t‐Lipo:Cy5.5 (40 µg in 100 µL PBS), or sonication followed by the same formulations after 5 min. No therapeutic siRNA treatment was administered before tissue collection, and the experiment was performed exclusively to assess formulation distribution. Sixteen hours later, the mice were euthanized and their brains and internal organs were promptly dissected. Fluorescence signals of Cy5.5 were captured using the Xenogen IVIS 200 imaging system (PerkinElmer) with a 1 ‐min exposure. The fluorescence intensity was quantified as the mean radiant efficiency [p/s/cm^2^/sr]/[mW/cm^2^] and normalized to the tumor volume, as determined by bioluminescence imaging.

### 4.17 Evaluation of BATUS for GBM Therapy

GL261 cells (5 × 10^4^) were injected stereotactically into the striatum of 8 weeks old C57BL/6 mice. After tumor formation was confirmed by bioluminescence imaging and analyzed by luciferase imaging, mice were randomized into six groups and treated with (1) Control (PBS), (2) Temozolomide (TMZ), (3) t‐Lipo:siRNA‐PD‐L1, (4) US + t‐Lipo:siRNA‐control, (5) US + non‐t‐Lipo:siRNA‐PDL1, or (6) US + t‐Lipo:siRNA‐PDL1 (n = 8). Mice received sonication followed by intravenous injection of formulations (40 µg in 100 µL PBS) every 3–4 days (on days 8, 11, 15, 18, 22, and 26 post‐implantation) for a total of six injections. Bioluminescence imaging was conducted weekly to assess tumor progression, and survival data were recorded.

### 4.18 Immuno/Histological analysis

One mouse from each group was euthanized after the final administration of the formulations. The brains, internal organs, and skin area surrounding the transducer (or not as a control) were harvested. The brains were fixed with 4% paraformaldehyde for 48h at 4°C, dehydrated with 30% sucrose solution, embedded in optimum cutting temperature (OCT)‐freeze medium (Sakura, CA, USA), and sectioned into 20 µm sections using a microtome (Leica Biosystems, IL, USA). Brain sections were stained with hematoxylin and eosin (H&E) to visualize the tumor area and were also stained with 6‐diamidino‐2phenylindole (DAPI) for nuclei, Alexa Fluor 488‐labeled anti‐PD‐L1 (1:100; v:v, eBioscience, CA, USA) and APC‐labeled anti‐CD8 (1:100; v:v, Thermo Fisher Scientific, USA) antibodies and observed using a fluorescence microscope (Discover ECHO Revolve, CA, USA).

Histological staining of the internal organs and skin was conducted on paraffin‐embedded sections. The tissues were collected, rinsed in PBS, and fixed with 10% formalin for 48h. Subsequently, the tissues were washed with PBS and placed in 70% ethanol solution until they were ready for paraffin embedding. Tissue blocks were sectioned into 5 µm‐thick slices. Internal organ sections were stained with H&E, while skin sections were stained with both H&E and MTS [73]. The stained slides were then examined using light microscopy (Discover ECHO Revolve, CA, USA).

### 4.19 Pig Studies

Large animal studies were approved by the John Hopkins University Animal Care and Use Committee and adhered to the guidelines established by the National Institutes of Health Guide for the Care and Use of Laboratory Animals. All animals were purchased from licensed purveyors. Female Yorkshire pigs weighing between 40–60 lbs were utilized. Animals were given a one‐week period of acclimatization during which veterinary technical checks were performed. All animals were fasted for a period of 12h prior to surgery. A preanesthetic combination of ketamine 20 mg/kg and xylazine 2 mg/kg was administered prior to intubation. All operative procedures were performed in Ross 450 using sterile technique. Once anesthetized, an endotracheal tube and intravenous catheter were placed in the marginal ear vein for delivery of intravenous fluids and drugs. Cefazolin (22 mg/kg IV) was administered approximately 10 min prior to the start of surgery and was repeated every 90 min intraoperatively. Heart rate, blood pressure, pulse oximetry, capnography, electrocardiography, respiratory rate, and rectal temperature were monitored continuously and recorded every 15–30 min throughout the procedure. Warming blankets were used to prevent hypothermia. Animals received a loading dose of fentanyl 0.05mg/kg i.v. followed by fentanyl CRI 0.03‐0.2mg/kg. During the procedure, jaw tone and palpebral reflex were used to confirm appropriate anesthesia. Anesthesia was provided using isoflurane at 0.5%–2.5% + 1–2 L 02/min and/or propofol 0.83‐L.66mg/kg i.v. The minimal alveolar concentration (MAC) was maintained at or below 0.5% for the duration of the procedure.

Once the pig was sedated and intubated, it was placed prone on the operating table. Scalp was prepared by trimming surface hair and prepping with Betadine and Chlorhexidine solution. Head was fixed onto the table using surgical tape. A point between the intersection of the external auditory meatuses and contralateral medial canthus of the eyes was used as center and a midline longitudinal 5 cm line was marked [74]. Incision was made along the marked line, and dissection carried out to the periosteum. Periosteum was elevated and scraped from the surface of the skull. Sagittal suture was visualized and a point 2 cm lateral to sagittal suture at midline was marked to avoid damaging the sagittal sinus.

Using a Midas Rex perforator (Medtronic Inc., Minneapolis, Minnesota, USA), a right‐sided burr hole was performed. The position of the burr hole was approximated to the motor cortex of the brain and further extended by Kerrison Rongeur until an appropriate amount of the dura was visualized. The device was positioned with the arrays over the dura. The lateral flaps of the biodegradable transducer (8 mm × 8 mm dimensions) were affixed onto the surrounding bone utilizing miniscrews. A Matrix plate (MatrixNEURO System, DePuy Synthes, Johnson&Johnson Medtech, Raynham, Massachusetts) was placed over the burr hole with the end of the device extending beyond the plate. Subsequently, a 3 cm horizontal incision was made above the existing midline incision through which the end of the device was tunneled out. The wound was then closed with sutures, and the device was covered with an appropriate dressing. Post‐operatively, the pig was extubated and monitored for any motor deficits. For the control group, burr hole was performed until an appropriate amount of dura was visualized, and the wound was then closed as explained above without transducer implantation.

After two weeks, the pigs were euthanized, and tissue samples were collected for histological analysis. Upon dissection of the cranium, the brain was carefully removed. Coronal sections were then obtained from regions of the dorsal frontoparietal cortex that directly interfaced with the implant. Sections of the spleen, liver, and kidney were also collected to assess a lack of systemic off‐target effects of biodegradable transducer.

Retrieved tissue samples were fixed in 10% neutral‐buffered formalin at 4°C for at least 48h. The samples were then submitted to the Johns Hopkins Oncology Tissue and Imaging Services core facility for further processing, embedding, sectioning, and staining. Briefly, samples were dehydrated, paraffin embedded, sectioned, and stained according to standard histological methods (Masson's trichrome or H&E staining). Stained slides were imaged using the Hamamatsu NanoZoomer. For a more detailed examination, the regions specified in the macroscopic views in Figure S5 were zoomed in and examined at the microscopic level and analyzed using NDP.view2 software.

### 4.20 Statistical Analysis

All statistical analyses were conducted using GraphPad Prism version 8 software. For comparisons involving two independent groups, an unpaired two‐tailed Student's *t*‐test was employed. To assess differences among multiple groups at a single time point including the terminal comparison of tumor growth, a one‐way ANOVA was utilized, followed by Sidak's multiple comparison test for pairwise group comparisons. To further evaluate longitudinal tumour‐growth dynamics, log10‐transformed bioluminescence data were additionally analyzed using a linear mixed‐effects model (REML), with treatment, time, and their interaction included as fixed effects and individual animals included as a random effect. To complement the terminal tumour‐burden analysis, cumulative tumour burden was also quantified as the area under the log10‐flux curve (AUC) and compared among groups using one‐way ANOVA. Kaplan−Meier curves and log‐rank (Mantel−Cox) tests were used to analyze survival data. For all analyses, a p‐value of less than 0.05 was considered statistically significant.

## Author Contributions

G.E.A., J.P. and T.D.N. conceived the study; G.E.A., J.P. and A.D. conducted the majority of the experimental work and data analyses; H.A. contributed to the design of formulation and assisted the flow cytometry analysis; XZ and HQT performed TEER experiment, N.B., H.A. and P.S. contributed to the animal experiments, S.K.K. helped to produce and test the biodegradable transducers, Z.L. helped for the electrospinning, T.T. performed the TEM analysis; C.T.G.N., Y.Z., and I.M. helped with immuno/histological analysis and evaluation, A.M., N.T., J.C.D. and T.D.N. designed the pig study, A.D.D., E.S., V.V., A.F.L, P.K., V.M.Q., B.B. and C.H. conducted pig study, G.E.A., J.P., A.D. and T.D.N. wrote/edited the manuscript with input from all authors; T.D.N. supervised the work and provided funding.

## Conflicts of Interest

T.D.N. is the cofounder of PiezoBioMembrane Inc. and SingleTimeMicroneedles Inc. The authors declare no other competing interests.

## Supporting information




**Supporting File 1**: advs76336‐sup‐0001‐SuppMat.docx.

**Supporting File 2**: advs76336‐sup‐0002‐MovieS1.mov.

## Data Availability

The main data supporting the results of this study are available within the paper and its Supplementary Information. The raw data is also published in the DRYAD. The remaining raw and analyzed datasets from the study are available for research purposes upon reasonable request from the corresponding author. Source data were provided in this study.

## References

[1] J. R. Wu , Y. Hernandez , K. F. Miyasaki , and E. J. Kwon , “Engineered nanomaterials that exploit blood‐brain barrier dysfunction for delivery to the brain,” Advanced Drug Delivery Reviews 197 (2023): 114820, 10.1016/j.addr.2023.114820. 37054953 PMC12834260

[2] L. Wang , M. Gu , X. Zhang , et al., “Recent Advances in Nanoenzymes Based Therapies for Glioblastoma: Overcoming Barriers and Enhancing Targeted Treatment,” Advanced Science 12 (2025): 2413367, 10.1002/advs.202413367. 39854126 PMC11905078

[3] Z. K. Englander , H.‐J. Wei , A. N. Pouliopoulos , et al., “Focused ultrasound mediated blood–brain barrier opening is safe and feasible in a murine pontine glioma model,” Scientific Reports 11 (2021): 6521, 10.1038/s41598-021-85180-y. 33753753 PMC7985134

[4] G. Shakya , M. Cattaneo , G. Guerriero , A. Prasanna , S. Fiorini , and O. Supponen , “Ultrasound‐responsive microbubbles and nanodroplets: A pathway to targeted drug delivery,” Advanced Drug Delivery Reviews 206 (2024): 115178, 10.1016/j.addr.2023.115178. 38199257

[5] P. Ghanouni , K. B. Pauly , W. J. Elias , et al., “Transcranial MRI‐Guided Focused Ultrasound: A Review of the Technologic and Neurologic Applications,” American Journal of Roentgenology 205 (2015): 150–159, 10.2214/AJR.14.13632. 26102394 PMC4687492

[6] N. Lipsman , Y. Meng , A. J. Bethune , et al., “Blood–brain barrier opening in Alzheimer's disease using MR‐guided focused ultrasound,” Nature Communications 9 (2018): 2336, 10.1038/s41467-018-04529-6. PMC6060168 30046032

[7] A. Carpentier , R. Stupp , A. M. Sonabend , et al., “Repeated blood–brain barrier opening With a nine‐emitter implantable ultrasound device in combination With carboplatin in recurrent glioblastoma: A phase I/II clinical trial,” Nature Communications 15 (2024): 15, 10.1038/s41467-024-45818-7. PMC10891097 38396134

[8] S. Guerin , S. A. M. Tofail , and D. Thompson , “Organic piezoelectric materials: Milestones and potential,” NPG Asia Materials 11 (2019): 10, 10.1038/s41427-019-0110-5.

[9] M. T. Chorsi , T. T. Le , F. Lin , et al., “Highly piezoelectric, biodegradable, and flexible amino acid nanofibers for medical applications,” Science Advances 9 (2023): eadg6075, 10.1126/sciadv.adg6075. 37315129 PMC10266740

[10] B. Wang , S. Hu , Y. Teng , et al., “Current advance of nanotechnology in diagnosis and treatment for malignant tumors,” Signal Transduction and Targeted Therapy 9 (2024): 200, 10.1038/s41392-024-01889-y. 39128942 PMC11323968

[11] R. De , S. Shi , and K. Kim , “Crossing the Blood–Brain Barrier With Molecularly Imprinted Polymeric Nanocarriers: An Emerging Frontier in Brain Disease Therapy,” Advanced Science 13 (2026): 17004, 10.1002/advs.202517004. PMC12866771 41316895

[12] R. Stupp , M. E. Hegi , W. P. Mason , et al., “Effects of radiotherapy With concomitant and adjuvant temozolomide versus radiotherapy alone on survival in glioblastoma in a randomised phase III study: 5‐year analysis of the EORTC‐NCIC trial,” The Lancet Oncology 10 (2009): 459–466, 10.1016/S1470-2045(09)70025-7. 19269895

[13] T. I. Janjua , P. Rewatkar , A. Ahmed‐Cox , et al., “Frontiers in the treatment of glioblastoma: Past, present and emerging,” Advanced Drug Delivery Reviews 171 (2021): 108–138, 10.1016/j.addr.2021.01.012. 33486006

[14] S. Zhou , Y. Huang , Y. Chen , et al., “Reprogramming systemic and local immune function to empower immunotherapy Against glioblastoma,” Nature Communications 14 (2023): 435, 10.1038/s41467-023-35957-8. PMC9880004 36702831

[15] A. Garcia‐Ruiz , P. Naval‐Baudin , M. Ligero , et al., “Precise enhancement quantification in post‐operative MRI as an indicator of residual tumor impact is associated With survival in patients With glioblastoma,” Scientific Reports 11 (2021): 11, 10.1038/s41598-020-79829-3. 33436737 PMC7804103

[16] H. You , S. Zhang , Y. Zhang , et al., “Engineered Bacterial Outer Membrane Vesicles‐Based Doxorubicin and CD47‐siRNA Co‐Delivery Nanoplatform Overcomes Immune Resistance to Potentiate the Immunotherapy of Glioblastoma,” Advanced Materials 37 (2025): 2418053, 10.1002/adma.202418053. 40035513

[17] D. Y. Lee , E. Im , D. Yoon , et al., “Pivotal role of PD‐1/PD‐L1 immune checkpoints in immune escape and cancer progression: Their interplay With platelets and FOXP3+Tregs related molecules, clinical implications and combinational potential With phytochemicals,” Seminars in Cancer Biology 86 (2022): 1033–1057, 10.1016/j.semcancer.2020.12.001. 33301862

[18] A. D. Waldman , J. M. Fritz , and M. J. Lenardo , “A guide to cancer immunotherapy: From T cell basic science to clinical practice,” Nature Reviews Immunology 20 (2020): 651–668, 10.1038/s41577-020-0306-5. PMC7238960 32433532

[19] X. Lin , K. Kang , P. Chen , et al., “Regulatory mechanisms of PD‐1/PD‐L1 in cancers,” Molecular Cancer 23 (2024): 108, 10.1186/s12943-024-02023-w. 38762484 PMC11102195

[20] H.‐T. Hsieh , H.‐C. Huang , C.‐W. Chung , et al., “CXCR4‐targeted nitric oxide nanoparticles deliver PD‐L1 siRNA for immunotherapy Against glioblastoma,” Journal of Controlled Release 352 (2022): 920–930, 10.1016/j.jconrel.2022.10.047. 36334859

[21] L. Galluzzi , T. A. Chan , G. Kroemer , J. D. Wolchok , and A. López‐Soto , “The hallmarks of successful anticancer immunotherapy,” Science Translational Medicine 10 (2018): aat7807, 10.1126/scitranslmed.aat7807. 30232229

[22] J. M. Colazo , M. C. Keech , V. Shah , et al., “siRNA conjugate With high albumin affinity and degradation resistance for delivery and treatment of arthritis in mice and guinea pigs,” Nature Biomedical Engineering 9 (2025): 1366–1383, 10.1038/s41551-025-01376-x. PMC12354308 40379798

[23] T. Jiang , Y. Qiao , W. Ruan , et al., “Cation‐Free siRNA Micelles as Effective Drug Delivery Platform and Potent RNAi Nanomedicines for Glioblastoma Therapy,” Advanced Materials 33 (2021): 2104779, 10.1002/adma.202104779. 34751990

[24] N. H. Tran , D. D. Nguyen , N. M. Nguyen , et al., “Dual‐Targeting Exosomes for Improved Drug Delivery in Breast Cancer,” Nanomedicine 18 (2023): 599–611, 10.2217/nnm-2022-0328. 37194929

[25] L. Zhu , H. Zhao , Z. Zhou , et al., “Peptide‐Functionalized Phase‐Transformation Nanoparticles for Low Intensity Focused Ultrasound‐Assisted Tumor Imaging and Therapy,” Nano Letters 18 (2018): 1831–1841, 10.1021/acs.nanolett.7b05087. 29419305

[26] F. Branco , J. Cunha , M. Mendes , C. Vitorino , and J. J. Sousa , “Peptide‐Hitchhiking for the Development of Nanosystems in Glioblastoma,” ACS Nano 18 (2024): 16359–16394, 10.1021/acsnano.4c01790. 38861272 PMC11223498

[27] C. E. Muolokwu , A. Gothwal , T. Kanekiyo , and J. Singh , “Synthesis and Characterization of Transferrin and Cell‐Penetrating Peptide‐Functionalized Liposomal Nanoparticles to Deliver Plasmid ApoE2 In Vitro and In Vivo in Mice,” Molecular Pharmaceutics 22 (2025): 229–241, 10.1021/acs.molpharmaceut.4c00870. 39665408 PMC11888121

[28] G. Erel‐Akbaba , H. Akbaba , O. Karaman , T. Tian , B. A. Tannous , and E. Turunc , “Rabies virus‐mimicking liposomes for targeted gene therapy in Alzheimer's disease,” International Journal of Pharmaceutics 668 (2025): 124962, 10.1016/j.ijpharm.2024.124962. 39592065

[29] S. Zha , H. Liu , H. Li , H. Li , K.‐L. Wong , and A. H. All , “Functionalized Nanomaterials Capable of Crossing the Blood–Brain Barrier,” ACS Nano 18 (2024): 1820–1845, 10.1021/acsnano.3c10674. 38193927 PMC10811692

[30] H. R. Li , M. Harb , J. E. Heath , J. S. Trippett , M. G. Shapiro , and J. O. Szablowski , “Engineering viral vectors for acoustically targeted gene delivery,” Nature Communications 15 (2024): 4924, 10.1038/s41467-024-48974-y. PMC11164914 38858354

[31] Y. Zhang , S. Saha , C. Dube , et al., 2024, 10.31225/osf.io/nakhd.

[32] A. Burgess , Y. Huang , W. Querbes , D. W. Sah , and K. Hynynen , “Focused ultrasound for targeted delivery of siRNA and efficient knockdown of Htt expression,” Journal of Controlled Release 163 (2012): 125–129, 10.1016/j.jconrel.2012.08.012. 22921802 PMC4010143

[33] Y. Guo , H. Lee , Z. Fang , et al., “Single‐cell analysis reveals effective siRNA delivery in brain tumors with microbubble‐enhanced ultrasound and cationic nanoparticles,” Science Advanced 7 (2021): abf7390. 10.1126/sciadv.abf7390 PMC8087400 33931452

[34] R. Ge , M. Chen , S. Wu , et al., “DNA nanoflower Oligo‐PROTAC for targeted degradation of FUS to treat neurodegenerative diseases,” Nature Communications 16 (2025): 4683, 10.1038/s41467-025-60039-2. PMC12092677 40394046

[35] A. Dasgupta , T. Sun , E. Rama , et al., “Transferrin Receptor‐Targeted Nonspherical Microbubbles for Blood–Brain Barrier Sonopermeation,” Advanced Materials 35 (2023): 2308150, 10.1002/adma.202308150. PMC11238272 37949438

[36] G. Kwak , A. Grewal , H. Slika , et al., “Brain Nucleic Acid Delivery and Genome Editing via Focused Ultrasound‐Mediated Blood–Brain Barrier Opening and Long‐Circulating Nanoparticles,” ACS Nano 18 (2024): 24139–24153, 10.1021/acsnano.4c05270. 39172436 PMC11792178

[37] E. L. Han , S. Tang , D. Kim , et al., “Peptide‐Functionalized Lipid Nanoparticles for Targeted Systemic mRNA Delivery to the Brain,” Nano Letters 25 (2025): 800–810, 10.1021/acs.nanolett.4c05186. 39688915

[38] K. K. Gill , A. Kaddoumi , and S. Nazzal , “PEG–lipid micelles as drug carriers: Physiochemical attributes, formulation principles and biological implication,” Journal of Drug Targeting 23 (2015): 222–231, 10.3109/1061186X.2014.997735. 25547369

[39] M. Kaurav , L. Gautam , and S. Minz , Advanced Pharmaceutical and Herbal Nanoscience for Targeted Drug Delivery Systems Part I, ed. S. Saraf , R. K. Sahu , and V. Dave , (BENTHAM SCIENCE PUBLISHERS, 2022), 133–168, 10.2174/97898150365101220101.

[40] T. Terada , J. A. Kulkarni , A. Huynh , et al., “Characterization of Lipid Nanoparticles Containing Ionizable Cationic Lipids Using Design‐of‐Experiments Approach,” Langmuir 37 (2021): 1120–1128, 10.1021/acs.langmuir.0c03039. 33439022

[41] T. Tian , R. Liang , G. Erel‐Akbaba , et al., “Immune Checkpoint Inhibition in GBM Primed With Radiation by Engineered Extracellular Vesicles,” ACS Nano 16 (2022): 1940–1953, 10.1021/acsnano.1c05505. 35099172 PMC9020451

[42] S. Khaledian , M. Dayani , A. Fatahian , R. Fatahian , and F. Martinez , “Efficiency of lipid‐based nano drug delivery systems in crossing the blood–brain barrier: A review,” Journal of Molecular Liquids 346 (2022): 118278, 10.1016/j.molliq.2021.118278.

[43] Y. Zhao , H. Zheng , X. Wang , et al., “Preparation and Biological Property Evaluation of Novel Cationic Lipid‐Based Liposomes for Efficient Gene Delivery,” Aaps Pharmscitech [Electronic Resource] 22 (2021): 22, 10.1208/s12249-020-01868-w. 33389222

[44] W. Y. Tong , M. Alnakhli , R. Bhardwaj , et al., “Delivery of siRNA in vitro and in vivo using PEI‐capped porous silicon nanoparticles to silence MRP1 and inhibit proliferation in glioblastoma,” Journal of Nanobiotechnology 16 (2018): 38, 10.1186/s12951-018-0365-y. 29653579 PMC5898074

[45] M. Van Woensel , N. Wauthoz , R. Rosière , et al., “Development of siRNA‐loaded chitosan nanoparticles targeting Galectin‐1 for the treatment of glioblastoma multiforme via intranasal administration,” Journal of Controlled Release 227 (2016): 71. 26902800 10.1016/j.jconrel.2016.02.032

[46] S. Antimisiaris , S. Mourtas , and K. Papadia , “Targeted si‐RNA With liposomes and exosomes (extracellular vesicles): How to unlock the potential,” International Journal of Pharmaceutics 525 (2017): 293–312, 10.1016/j.ijpharm.2017.01.056. 28163221

[47] S. Abuhelal , M. N. Centelles , M. Wright , A. J. Mason , and M. Thanou , “Development of Cationic Lipid LAH4‐L1 siRNA Complexes for Focused Ultrasound Enhanced Tumor Uptake,” Molecular Pharmaceutics 20 (2023): 2341–2351, 10.1021/acs.molpharmaceut.2c00909. 36989421 PMC10155207

[48] J. E. Francis , I. Skakic , C. Dekiwadia , et al., “Solid Lipid Nanoparticle Carrier Platform Containing Synthetic TLR4 Agonist Mediates Non‐Viral DNA Vaccine Delivery,” Vaccines 8 (2020): 551, 10.3390/vaccines8030551. 32967285 PMC7563538

[49] G. Rassu , E. Soddu , A. M. Posadino , et al., “Nose‐to‐brain delivery of BACE1 siRNA loaded in solid lipid nanoparticles for Alzheimer's therapy,” Colloids and Surfaces B: Biointerfaces 152 (2017): 296–301, 10.1016/j.colsurfb.2017.01.031. 28126681

[50] X. Liang , X. Li , J. Chang , Y. Duan , and Z. Li , “Properties and Evaluation of Quaternized Chitosan/Lipid Cation Polymeric Liposomes for Cancer‐Targeted Gene Delivery,” Langmuir 29 (2013): 8683–8693, 10.1021/la401166v. 23763489

[51] J. Ju , M.‐L. Huan , N. Wan , H. Qiu , S.‐Y. Zhou , and B.‐L. Zhang , “Novel Cholesterol‐Based Cationic Lipids as Transfecting Agents of DNA for Efficient Gene Delivery,” International Journal of Molecular Sciences 16 (2015): 5666–5681, 10.3390/ijms16035666. 25768346 PMC4394498

[52] X. Ma , F. Wu , C. Peng , H. Chen , D. Zhang , and T. Han , “Exploration of mRNA nanoparticles based on DOTAP Through optimization of the helper lipids,” Biotechnology Journal 18 (2023): 2300123, 10.1002/biot.202300123. 37545293

[53] G. Erel‐Akbaba , L. A. Carvalho , T. Tian , et al., “Radiation‐Induced Targeted Nanoparticle‐Based Gene Delivery for Brain Tumor Therapy,” ACS Nano 13 (2019): 4028–4040, 10.1021/acsnano.8b08177. 30916923 PMC7104714

[54] Y. Liu , G. Dzidotor , T. T. Le , et al., “Exercise‐induced piezoelectric stimulation for cartilage regeneration in rabbits,” Science Translational Medicine 14 (2022): eabi7282, 10.1126/scitranslmed.abi7282. 35020409

[55] J. T. Miyauchi , D. Chen , M. Choi , et al., “Ablation of Neuropilin 1 From glioma‐associated microglia and macrophages slows tumor progression,” Oncotarget 7 (2016): 9801–9814, 10.18632/oncotarget.6877. 26755653 PMC4891085

[56] L. Chen , W. Miao , X. Tang , et al., “Inhibitory Effect of Neuropilin‐1 Monoclonal Antibody (NRP‐1 MAb) on Glioma Tumor in Mice,” Journal of Biomedical Nanotechnology 9 (2013): 551–558, 10.1166/jbn.2013.1623. 23621013

[57] N. Ahmad , G. Kiriako , J. Saliba , K. Abla , M. El‐Sabban , and R. Mhanna , “Engineering a 3D Biomimetic Peptides Functionalized‐Polyethylene Glycol Hydrogel Model Cocultured With Endothelial Cells and Astrocytes: Enhancing In Vitro Blood–Brain Barrier Biomimicry,” Molecular Pharmaceutics 21 (2024): 4664–4672, 10.1021/acs.molpharmaceut.4c00599. 39133897 PMC11372828

[58] L. Jena , E. McErlean , and H. McCarthy , “Delivery Across the blood‐brain barrier: Nanomedicine for glioblastoma multiforme,” Drug Delivery and Translational Research 10 (2020): 304–318, 10.1007/s13346-019-00679-2. 31728942 PMC7066289

[59] M. Cattaneo , G. Guerriero , G. Shakya , et al., “Cyclic jetting enables microbubble‐mediated drug delivery,” Nature Physics 21 (2025): 590–598, 10.1038/s41567-025-02785-0. 40248569 PMC11999868

[60] W. A. Banks , E. M. Rhea , M. J. Reed , and M. A. Erickson , “The penetration of therapeutics Across the blood‐brain barrier: Classic case studies and clinical implications,” Cell Reports Medicine 5 (2024): 101760, 10.1016/j.xcrm.2024.101760. 39383873 PMC11604479

[61] I. B. Belyaev , O. Y. Griaznova , A. V. Yaremenko , S. M. Deyev , and I. V. Zelepukin , “Beyond the EPR effect: Intravital microscopy analysis of nanoparticle drug delivery to tumors,” Advanced Drug Delivery Reviews 219 (2025): 115550, 10.1016/j.addr.2025.115550. 40021012

[62] B. Dai , N. Qi , J. Li , and G. Zhang , “Temozolomide combined With PD‐1 Antibody therapy for mouse orthotopic glioma model,” Biochemical and Biophysical Research Communications 501 (2018): 871–876, 10.1016/j.bbrc.2018.05.064. 29758196

[63] D. Saha , S. D. Rabkin , and R. L. Martuza , “Temozolomide antagonizes oncolytic immunovirotherapy in glioblastoma,” Journal for ImmunoTherapy of Cancer 8 (2020): 000345, 10.1136/jitc-2019-000345. PMC7252967 32457126

[64] D. Lin , Z. Wang , W. Long , et al., “Nanosonosensitizer‐Augmented Sono‐Immunotherapy for Glioblastoma by Non‐Invasive Opening of the Blood–Brain Barrier,” Advanced Functional Materials 33 (2023): 2209219, 10.1002/adfm.202209219.

[65] T. Shan , W. Wang , M. Fan , et al., “Effective glioblastoma immune sonodynamic treatment mediated by macrophage cell membrane cloaked biomimetic nanomedicines,” Journal of Controlled Release 370 (2024): 866–878, 10.1016/j.jconrel.2024.04.043. 38685386

[66] T. Tian , H.‐X. Zhang , C.‐P. He , et al., “Surface functionalized exosomes as targeted drug delivery vehicles for cerebral ischemia therapy,” Biomaterials 150 (2018): 137–149, 10.1016/j.biomaterials.2017.10.012. 29040874

[67] M. Silginer , M. Weller , U. Ziegler , and P. Roth , “Integrin inhibition promotes atypical anoikis in glioma cells,” Cell death & disease 5 (2014): 1012. 10.1038/cddis.2013.543 PMC4040659 24457956

[68] D.‐W. Lu , C.‐Y. Shiau , C.‐H. Chiang , and M.‐K. Y. Chen , “Efficient downregulation of VEGF in retinal pigment epithelial cells by integrin ligand‐labeled liposome‐mediated siRNA,” IJN 8 (2013): 2613. 23901275 10.2147/IJN.S39622 PMC3726441

[69] G. Kumar , P. Mullick , S. B. Andugulapati , et al., “Trastuzumab‐conjugated liposomes for co‐delivery of paclitaxel and anti‐abcb1 siRNA in HER2‐positive breast cancer: In vitro and in vivo evaluations,” Journal of Drug Delivery Science and Technology 95 (2024): 105614, 10.1016/j.jddst.2024.105614.

[70] E. J. Curry , T. T. Le , R. Das , et al., “Biodegradable nanofiber‐based piezoelectric transducer,” Proceedings of the National Academy of Sciences 117 (2020): 214–220, 10.1073/pnas.1910343117. PMC6955346 31871178

[71] S. Garofalo , G. D'Alessandro , G. Chece , et al., “Enriched environment reduces glioma growth Through immune and non‐immune mechanisms in mice,” Nature Communications 6 (2015): 6623, 10.1038/ncomms7623. PMC4389244 25818172

[72] Z. Li , S. Jiang , J. Wang , et al., “Peptide‐drug conjugates repolarize glioblastoma‐associated macrophages to resensitize chemo‐immunotherapy of glioblastoma,” Science Advances 11 (2025): adr8841, 10.1126/sciadv.adr8841. PMC11740939 39823328

[73] G. Erel‐Akbaba , H. Akbaba , E. Keselik , S. A. Bahceci , Z. Senyigit , and T. K. Temiz , “Octaarginine functionalized nanoencapsulated system: In vitro and in vivo evaluation of bFGF loaded formulation for wound healing,” Journal of Drug Delivery Science and Technology 71 (2022): 103343, 10.1016/j.jddst.2022.103343.

[74] A. Kinaci , K. Vaessen , S. Redegeld , A. Van Der Zwan , and T. P. C. Van Doormaal , “Minimizing complications in a porcine survival craniotomy model,” Laboratory Animals 55 (2021): 435–442, 10.1177/00236772211009435. 34018879

