# Supplementary material for: Biodegradable Acoustic Targeting for Ultrasound‐Supported Gene Therapy (BATUS) in Glioblastoma
Source: Adv Sci (Weinh). 2026 Jun 29;13(53):e76336. doi: 10.1002/advs.76336 (PMC13336871; doi:10.1002/advs.76336)
Supplement: Supplementary file 1 [file ADVS-13-e76336-s001.docx]

**Supplementary Information**

**Biodegradable Acoustic Targeting for Ultrasound-Supported Gene Therapy (BATUS) in Glioblastoma**

**Corresponding author: Thanh D. Nguyen,** [**nguyentd@uconn.edu**](mailto:nguyentd@uconn.edu)

**Supplementary Fig. 1.** ***In vitro* cytotoxicity and hemocompatibility assessment of the developed liposomes**. **(a)** Cell viability of GL261 and U87 glioma cell lines treated with Lipo and t-Lipo formulations; Data represent mean± SD (n = 4) **(b)** Hemolysis percentage of mouse erythrocytes treated with t-Lipo:siRNA complexes. Data are presented as the mean ± SD, with hemolysis rates below the 5% threshold required by ISO 10993-4.

**Supplementary Fig. 2. TEER recovery and endothelial viability after device-mediated ultrasound exposure.** **(a)** TEER of blank bEnd.3 endothelial layers was measured before and after ultrasound exposure to assess endothelial barrier integrity and recovery. **(b)** Cell viability of bEnd.3 endothelial cells after ultrasound exposure was assessed using a CCK-8 assay. Data represent mean ± SD (n = 4). ***P < 0.001; ns, not significant.

**Supplementary Fig. 3.** ***In vivo* application of BATUS for brain cancer therapy in C57BL/6 mice with orthotopic glioblastoma**. **(a-c)** Surgical procedures for establishing the orthotopic glioblastoma xenograft model: **(a)** Anesthetized mouse positioned for stereotactic injection, **(b)** craniotomy performed to expose the skull, **(c)** injection of GL261cells into the brain via a 30-gauge Hamilton syringe at coordinates 2.5 mm lateral and 0.5 mm anterior to bregma, to a depth of 2.5 mm. **(d-f)** Biodegradable transducer implantation process: **(d)** biodegradable glycine-PCL transducer positioned in the craniotomy defect; **(e)** securing the transducer with surgical tools and closing the incision; **(f)** A bolus of 30μl ultrasound contrast agent injected into the animal through the retro-orbital route before sonication. The device was driven by a power amplifier and function generator to generate ultrasound, followed by intravenous injection of t-LiposiRNA.

**Supplementary Fig. 4.** **Safety evaluation of the BATUS technology in an orthotopic glioblastoma xenograft model in terms of skin irritation and body weight**. **(a)** Histological analysis of the skin surrounding the implantation site in C57BL/6 mice, comparing the control (no device) and implanted device. Scale bars = 110 μm. **(b)** Body weight monitoring of mice treated with control, temozolomide, t-Lipo:siRNA-PDL1, US + t-Lipo:siRNA-control, US + non-t-Lipo:siRNA-PDL1, and US + t-Lipo:siRNA-PDL1 over 28 days post-implantation. Data represent mean ± SD (n=8), showing no significant weight loss across treatment groups, confirming the absence of systemic toxicity.

**Supplementary Fig. 5. Quantitative analysis of PD-L1 and CD8 immunofluorescence in GBM tumor sections.** Quantification of PD-L1 and CD8 fluorescence signals from stained brain tumor sections. Data presented as mean ± SD (n = 5). **P < 0.01, ***P < 0.001, ****P < 0.0001.

**Supplementary Fig. 6.** **Schematic representation of brain tissue sampling for histological analysis in the Yorkshire pig study.** The diagram illustrates the process of collecting samples from both the implantation hemisphere and the opposite hemisphere following transducer implantation. Samples are taken using a biopsy tool and subsequently fixed for histological examination to assess local tissue response and biocompatibility.

**Supplementary Fig. 7.** **Macroscopic overview of tissue regions for** **histological safety evaluation of BATUS technology in a Yorkshire pig model, with boxed areas for microscopic analysis**. **(a)** H&E and Masson’s Trichrome-stained brain sections from implantation (regions 1 and 2) and control hemispheres (regions 1 and 2), 14 days post-transducer implantation. Scale bars = 500 μm. **(b)** H&E and Masson’s Trichrome-stained sections of liver, spleen, and kidney from implantation and control groups. Scale bars = 110 μm

**Movie S1.** Freely moving the mouse with an implanted biodegradable device during the treatment period via BATUS technology
